# Supplementary material for: Toward an optimal contraception dosing strategy
Source: PLoS Comput Biol. 2023 Apr 13;19(4):e1010073. doi: 10.1371/journal.pcbi.1010073 (PMC10101497; doi:10.1371/journal.pcbi.1010073)
Supplement: S1 Text — This file presents the data used in our study, model parameters, standard deviation of model parameters, result of the sensitivity analysis performed on the model, effect of various weights on the optimal control results, optimal control results from different forms of the objective function, and computation time and optimal cost for the optimal control simulations. (PDF) [file pcbi.1010073.s001.pdf]

S1 - Supporting Information

For: *Toward an optimal contraception dosing strategy* (2023), Brenda Lyn A. Gavina, Aurelio A. de los Reyes V, Mette S. Olufsen, Suzanne Lenhart, Johnny T. Ottesen, PLoS Computational Biology.

Contents

1 Data and standard deviation of model parameters **2**

2 Sensitivity Analysis **6**

3 Optimal exogenous hormones for different weights **7**

4 Model output for variants of the objective function **8**

5 Computation time and optimal cost **11**

1 Data and standard deviation of model parameters

This section presents the data used in our study, model parameters, and the standard deviation of model parameters.

Table A. Mean levels of pituitary and ovarian hormones. Data extracted from Fig 1 in [1] using the software DigitizeIt version 2.5 [2], comprise mean levels of E_2, P_4, $InhA$, LH, and FSH taken from 23 normally cycling women.

**Day**	$\mathbf{E_2}$ [pg/mL]	$\mathbf{P_4}$ [ng/mL]	**InhA** [IU/mL]	**LH** [IU/L]	**FSH** [IU/L]
1	51	1.1	1.0	12	11.4
2	55	0.6	1.1	14	11.6
3	53	0.6	1.0	15	11.7
4	59	0.6	1.1	14	12.4
5	60	0.6	1.1	17	12.6
6	62	0.6	1.0	17	11.3
7	66	0.6	1.1	19	12.1
8	72	0.6	1.2	18	11.3
9	95	0.6	1.7	17	10.0
10	119	0.6	2.3	17	8.7
11	138	0.6	3.2	17	8.6
12	188	0.6	4.5	25	8.2
13	237	0.7	7.4	50	10.4
14	215	1.2	9.3	123	19.6
15	127	2.0	7.7	41	12.1
16	91	5.0	8.1	22	9.2
17	102	8.9	10.1	20	8.7
18	119	11.2	8.9	20	8.6
19	140	15.6	9.5	18	7.4
20	133	17.3	11.5	16	7.2
21	152	17.9	9.1	12	6.1
22	142	17.2	8.7	9	5.4
23	140	14.4	7.5	11	5.2
24	155	12.6	6.6	10	5.4
25	133	10.3	5.7	11	5.3
26	114	8.1	4.1	11	6.1
27	70	4.3	2.2	11	6.7
28	55	1.9	1.7	11	8.3

Table B. Model parameter symbols, descriptions, values, units, and references.

Parameter	Description	Value	Unit	Reference
k_{LH}	LH release to the blood rate	0.9661	day^{-1}	estimated
α_{LH}	LH clearance rate	14.0	day^{-1}	[3]
$V_{0,LH}$	LH non-induced max synthesis rate	550.03	$IUday^{-1}$	estimated
$V_{1,LH}$	LH max synthesis rate	3329.19	$IUday^{-1}$	estimated
km_{LH}	E_2 value at half-saturation	136.05	$pgmL^{-1}$	estimated
	Constant for inhibition of			
$ki_{LH,P}$	LH synthesis	6.78	$ngmL^{-1}$	estimated
$c_{LH,E}$	LH release to the blood	0.0060	$mLpg^{-1}$	estimated
$c_{FSH,E}$	FSH release	0.0151	$\left(\frac{mL}{pg}\right)^2$	estimated
$Ki_{FSH,Inh}$	FSH synthesis	16.83	$IUmL^{-1}$	estimated
w^*	FSH synthesis	9.21	ng/mL	estimated
q^*	RcF	5.11	ng/mL	estimated
	constant for stimulation of			
$c_{LH,P}$	LH release to the blood	1.98	$mLng^{-1}$	estimated
$c_{FSH,P}$	FSH release	52.31	$mLng^{-1}$	estimated
V_{FSH}	FSH maximal synthesis rate	294.90	$IUday^{-1}$	estimated
α_{FSH}	FSH clearance rate	8.21	day^{-1}	[3]
k_{FSH}	FSH release to the blood rate	14.59	day^{-1}	estimated
τ	Time scale controlling Inh concentration and FSH synthesis	1.5	$days$	[3]
v	Blood volume	2.50	L	[3]
	Follicles transition factor from			
b	inactive to RcF	0.0453	Lμg(IUday)$^{-1}$	estimated
c_2	RcF to GrF	0.0577	$(L/IU)^{\alpha}/day$	estimated
c_3	GrF to $DomF$	0.0170	$L/(IUday)$	estimated
c_4	$DomF$ to ovulation	1.14	$(L/IU)^{\gamma}/day$	estimated
d_1	Sc_1 to Sc_2	0.7537	day^{-1}	estimated
d_2	Sc_2 to Lut_1	0.6866	day^{-1}	estimated
k_1	Lut_1 to Lut_2	0.6699	day^{-1}	estimated
k_2	Lut_2 to Lut_3	0.6388	day^{-1}	estimated
k_3	Lut_3 to Lut_4	0.9191	day^{-1}	estimated
c_1	RcF stage follicles growth factor	0.1036	$LIU^{-1}day^{-1}$	estimated
k_4	Lut_4 stage degradation factor for corpus lutea	1.88	day^{-1}	estimated

* added to the Margolskee model.

Model parameter symbols, descriptions, values, units, and references.

**Parameter**	**Description**	**Value**	**Unit**	**Reference**
α	LH exponent for promotion from RcF to GrF	0.9505		estimated
γ	LH exponent for transition from $DomF$ to ovulation	0.1615		estimated
e_0	Min E_2 level	57.60	$pgmL^{-1}$	estimated
h_0	Min Inh level	0.6606	$IUmL^{-1}$	estimated
	Contribution factor of			
e_1	GrF to E_2	0.0269	L^{-1}	estimated
e_2	$DomF$ to E_2	0.4196	L^{-1}	estimated
e_3	Lut_4 to E_2	0.4923	L^{-1}	estimated
p_1	Lut_3 to P_4	0.0032	kL^{-1}	estimated
p_2	Lut_4 to P_4	0.1188	kL^{-1}	estimated
h_1	$DomF$ to Inh	0.0193	IU$mL^{-1}\mu g^{-1}$	estimated
h_2	Lut_3 to Inh	0.0159	$IUmL^{-1}\mu g^{-1}$	estimated
h_3	Lut_4 to Inh	0.0119	$IUmL^{-1}\mu g^{-1}$	estimated
h_0	Min Inh level	0.6606	$IUmL^{-1}$	estimated

* added to the Margolskee model.

Table C. Standard deviation of model parameters (Par) and initial conditions (IC). Mean and standard deviation of parameters and initial conditions obtained from bootstrapping with 1000 random initial guesses.

Par/IC	Value	Unit	Mean ± Standard deviation
k_{LH}	0.9661	day^{-1}	1.0212 ± 0.2854
$V_{0,LH}$	550.03	IUday^{-1}	627.08 ± 28.44
$V_{1,LH}$	3329.19	IUday^{-1}	3499.11 ± 841.25
km_{LH}	136.05	pgmL^{-1}	164.52 ± 16.28
$ki_{LH,P}$	6.78	ngmL^{-1}	7.55 ± 2.01
$c_{LH,E}$	0.0060	mLpg^{-1}	0.0053 ± 0.0025
$c_{LH,P}$	1.98	mLng^{-1}	2.11 ± 0.83
V_{FSH}	294.90	IUday^{-1}	274.55 ± 16.36
k_{FSH}	14.59	day^{-1}	14.95 ± 8.88
$c_{FSH,E}$	0.0151	$\left(\frac{mL}{pg}\right)^2$	0.0147 ± 0.0048
$Ki_{FSH,Inh}$	16.83	IUmL^{-1}	17.79 ± 7.26
$c_{FSH,P}$	52.31	mLng^{-1}	44.89 ± 24.26
b	0.0453	$\mathrm{L}\mu\mathrm{g}(\mathrm{IUday})^{-1}$	0.0550 ± 0.0227
c_1	0.1036	$\mathrm{LIU}^{-1}\mathrm{day}^{-1}$	0.1230 ± 0.0202
c_2	0.0577	$(\mathrm{L/IU})^{\alpha}/\mathrm{day}$	0.0502 ± 0.0185
c_3	0.0170	$\mathrm{L}/(\mathrm{IUday})$	0.0142 ± 0.0053
c_4	1.14	$(\mathrm{L/IU})^{\gamma}/\mathrm{day}$	0.91 ± 0.36
d_1	0.7537	day^{-1}	0.7758 ± 0.1288
d_2	0.6866	day^{-1}	0.7558 ± 0.1225
k_1	0.6699	day^{-1}	0.7393 ± 0.1153
k_2	0.6388	day^{-1}	0.6503 ± 0.0960
k_3	0.9191	day^{-1}	0.7092 ± 0.1237
k_4	1.88	day^{-1}	1.59 ± 0.34
α	0.9505		1.0163 ± 0.1320
γ	0.1615		0.2337 ± 0.1634
e_0	57.60	pg/mL	52.24 ± 3.77
e_1	0.0269	L^{-1}	0.0638 ± 0.0433
e_2	0.4196	L^{-1}	0.4265 ± 0.1468
e_3	0.4923	L^{-1}	0.5991 ± 0.1310
p_1	0.0032	kL^{-1}	0.0025 ± 0.0013
p_2	0.1188	kL^{-1}	0.0964 ± 0.0225
h_0	0.6606	IUmL^{-1}	0.7096 ± 0.0958
h_1	0.0193	$\mathrm{IUmL}^{-1}\mu\mathrm{g}^{-1}$	0.0246 ± 0.0069
h_2	0.0159	$\mathrm{IUmL}^{-1}\mu\mathrm{g}^{-1}$	0.0147 ± 0.0052
h_3	0.0119	$\mathrm{IUmL}^{-1}\mu\mathrm{g}^{-1}$	0.0163 ± 0.0077
w	9.21	ng/mL	11.87 ± 3.64
q	5.11	ng/mL	6.06 ± 2.06
$RP_{LH}(0)$	167.57	IU	155.52 ± 66.54
$LH(0)$	11.81	IU/L	11.02 ± 3.01
$RP_{FSH}(0)$	14.48	IU	13.41 ± 11.87
$FSH(0)$	11.41	IU/L	9.86 ± 3.45
$RcF(0)$	2.10	ng	2.18 ± 0.81
$GrF(0)$	4.12	ng	5.23 ± 5.78
$DomF(0)$	0.46	ng	0.48 ± 0.61
$Ov_1(0)$	1.06	ng	1.19 ± 1.46
$Ov_2(0)$	1.67	ng	2.26 ± 4.12
$Lut_1(0)$	4.16	ng	6.28 ± 7.59
$Lut_2(0)$	13.03	ng	18.44 ± 11.58
$Lut_3(0)$	16.48	ng	14.79 ± 5.93
$Lut_4(0)$	10.29	ng	9.54 ± 2.69

2 Sensitivity Analysis

This part shows the result of the sensitivity analysis performed on the model.

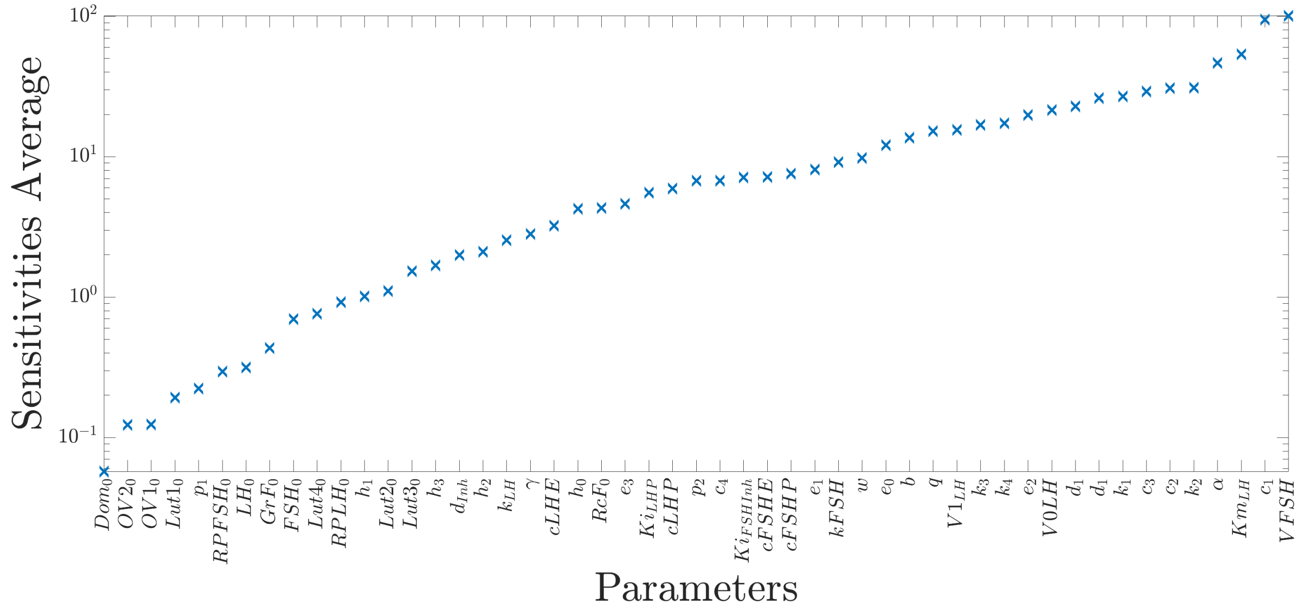

Fig A. Sensitive parameters. Sensitivity analysis reveals parameters that affect the model output most.

3 Optimal exogenous hormones for different weights

This section presents the effect of various weights on the optimal control results.

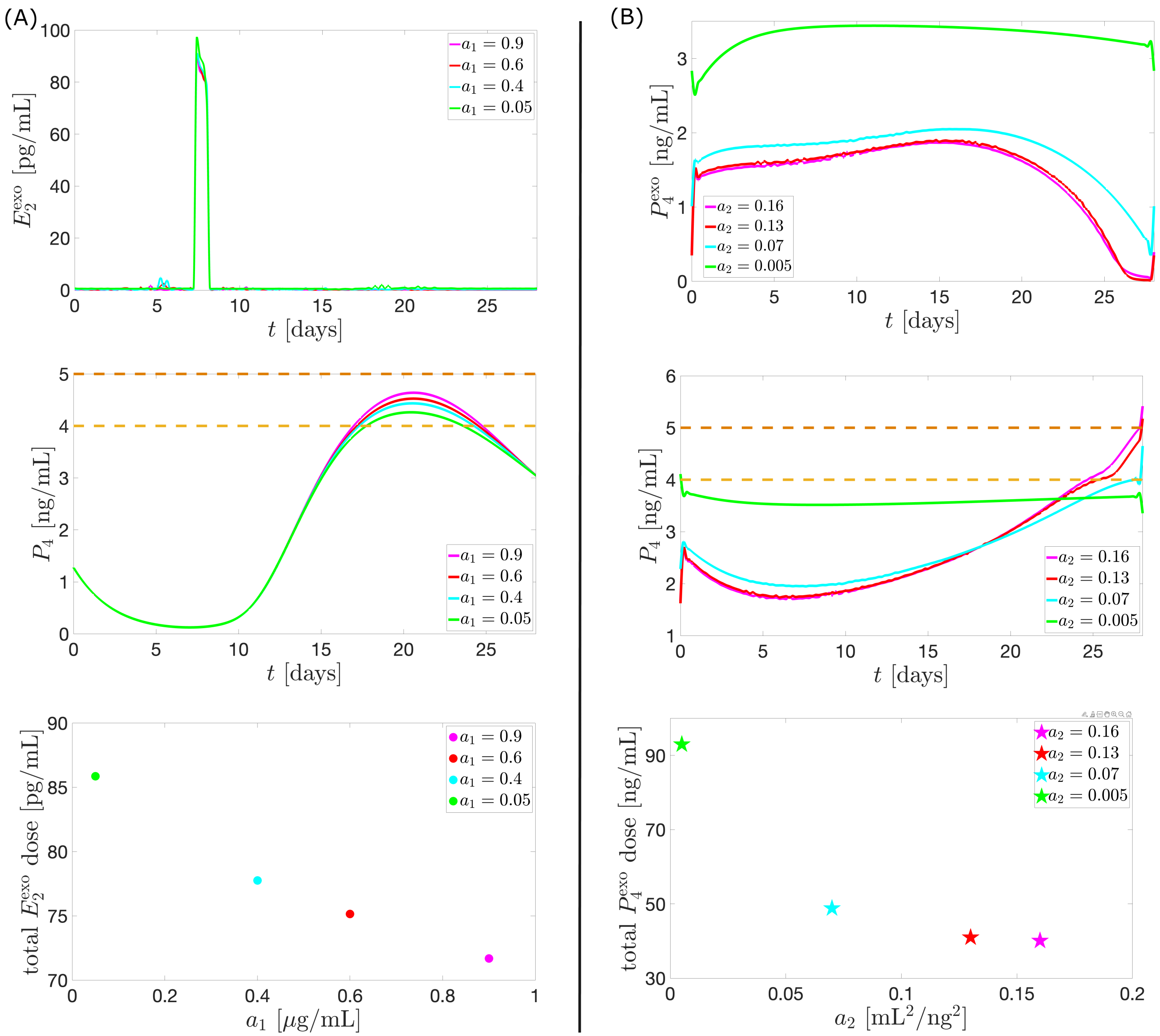

Fig B. Varying weight parameters in monotherapy. Panel (A) shows the effect of varying the weight parameter a_1 in estrogen monotherapy on the optimal E_2^{exo}, and subsequently on the P_4 level and total exogenous estrogen dose. Panel (B) presents the effect of changing a_2 in progesterone monotherapy on optimal P_4^{exo}, on P_4 concentration, and total exogenous progesterone dose.

4 Model output for variants of the objective function

Here we show the optimal control results from different forms of the objective function.

1. Optimal exogenous estrogen and progesterone if the objective function is

$$\int_0^{28} \left((P_4(t) - 4)^2 + a_1 u_1 + a_2 u_2\right) dt.$$

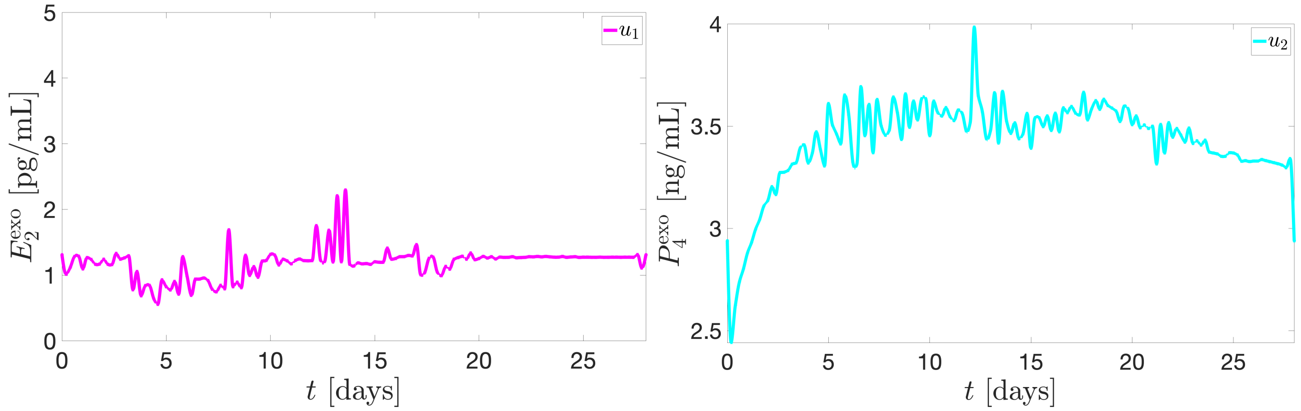

Fig C. Linear third term in objective function with $a_1 = 0.4$ and $a_2 = 0.7$. Optimal exogenous estrogen (on the left) and optimal exogenous progesterone (on the right) if the objective function is $\int_0^{28} \left((P_4(t) - 4)^2 + 0.4u_1 + 0.7u_2\right) dt$.

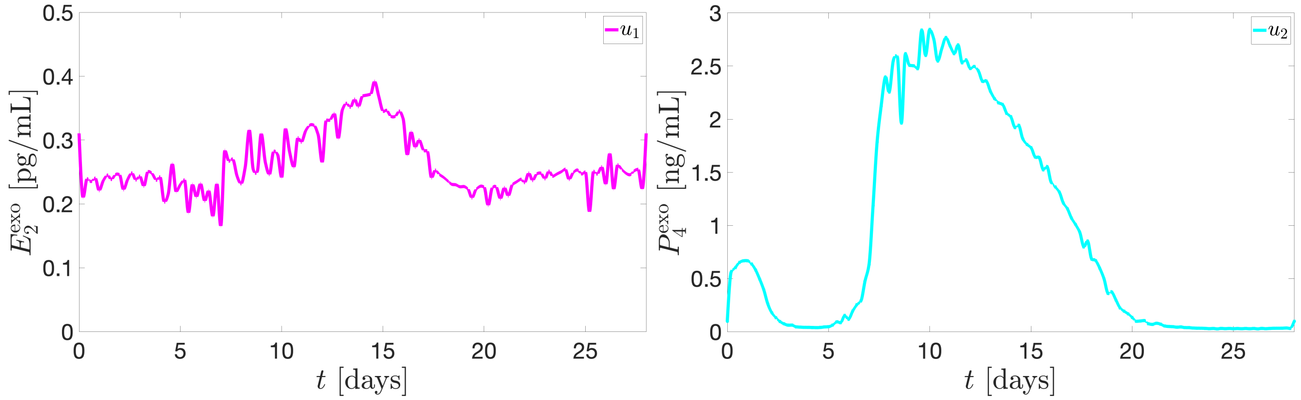

Fig D. Linear third term in objective function with $a_1 = 0.4$ and $a_2 = 2$. Optimal exogenous estrogen (on the left) and optimal exogenous progesterone (on the right) if the objective function is $\int_0^{28} \left((P_4(t) - 4)^2 + 0.4u_1 + 2u_2\right) dt$.

2. Optimal exogenous estrogen and progesterone if the objective function is

$$\int_0^{28} \left((P_4(t)-4)^2 + a_1 u_1 + a_2 u_2^2\right) dt.$$

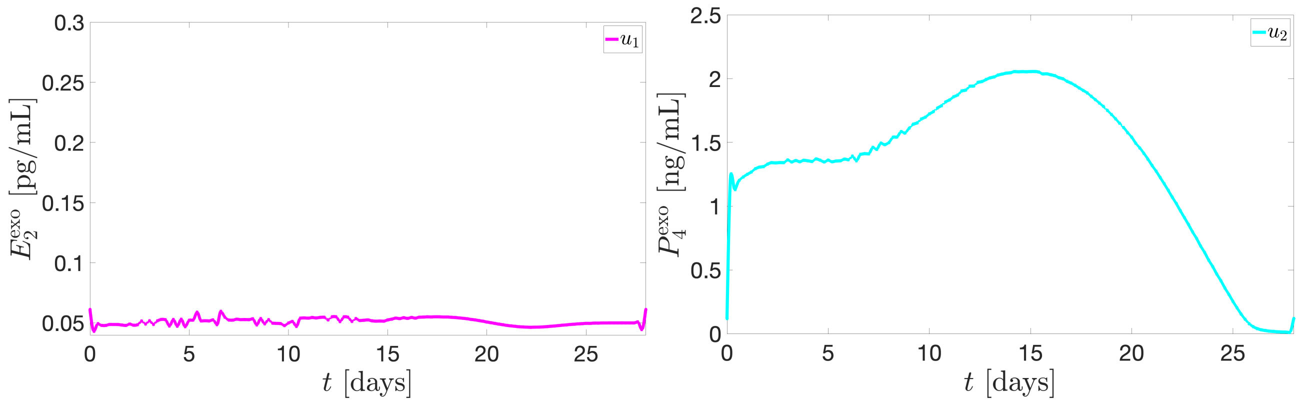

Fig E. Quadratic third term in objective function with $a_1 = 0.4$ and $a_2 = 0.7$. Optimal exogenous estrogen (on the left) and optimal exogenous progesterone (on the right) if the objective function is $\int_0^{28} \left((P_4(t)-4)^2 + 0.4u_1 + 0.7u_2^2\right) dt$.

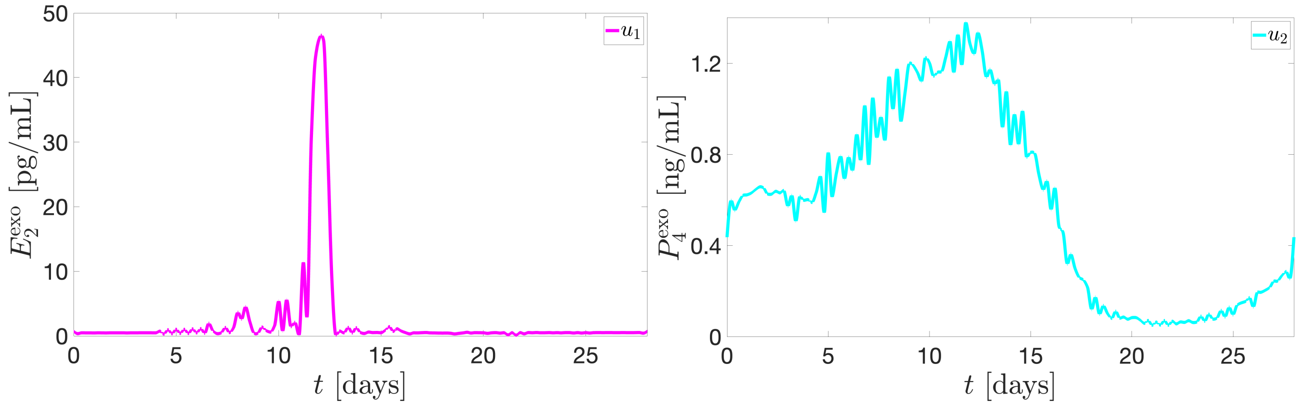

Fig F. Quadratic third term in objective function with $a_1 = 0.2$ and $a_2 = 2.5$. Optimal exogenous estrogen (on the left) and optimal exogenous progesterone (on the right) if the objective function is $\int_0^{28} \left((P_4(t)-4)^2 + 0.2u_1 + 2.5u_2^2\right) dt$.

3. Optimal exogenous estrogen and progesterone if the objective function is

$$\int_0^{28} \left((P_4(t) - 4)^2 + a_1 u_1 + a_2 u_2^3\right) dt.$$

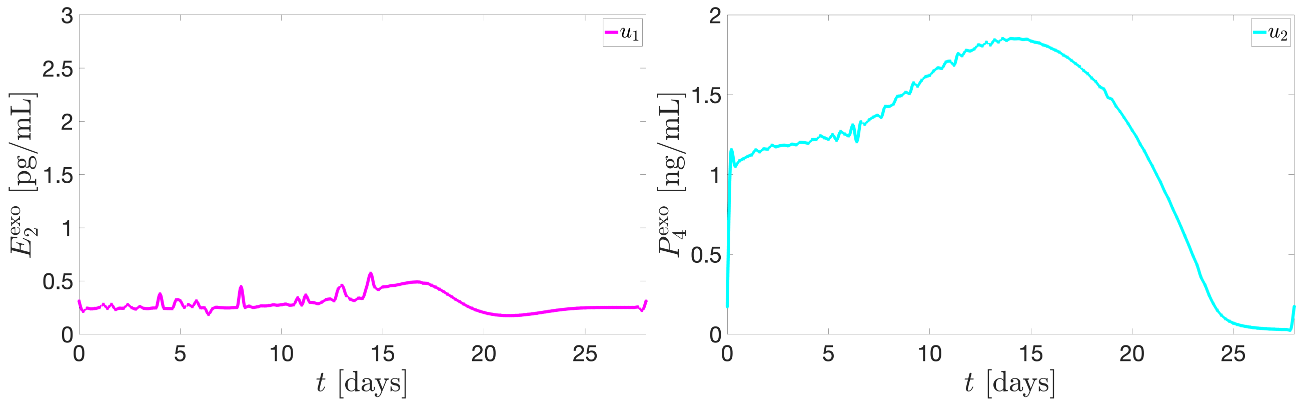

Fig G. Cubic third term in objective function with $a_1 = 0.4$ **and** $a_2 = 0.7$**.** Optimal exogenous estrogen (on the left) and optimal exogenous progesterone (on the right) if the objective function is $\int_0^{28} \left((P_4(t) - 4)^2 + 0.4u_1 + 0.7u_2^3\right) dt$.

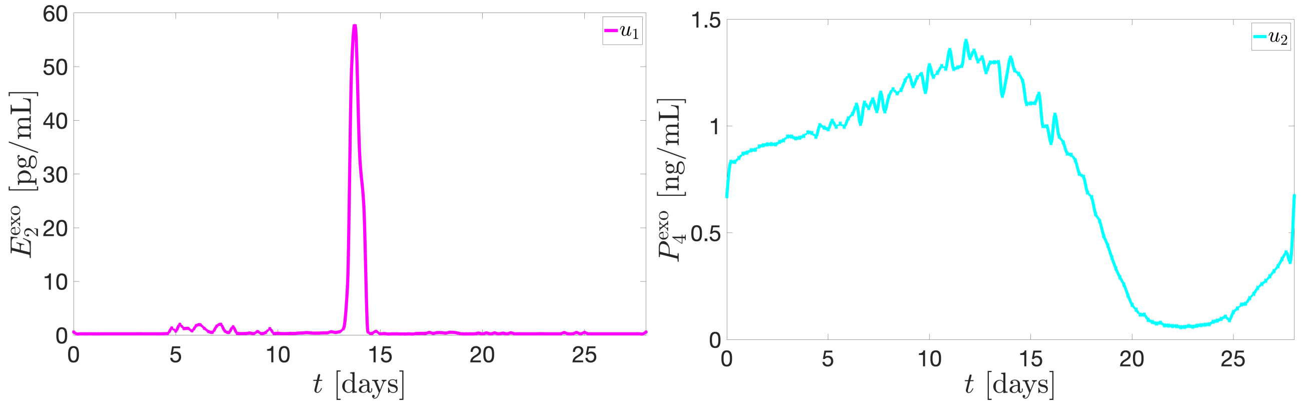

Fig H. Cubic third term in objective function with $a_1 = 0.4$ **and** $a_2 = 1.5$**.** Optimal exogenous estrogen (on the left) and optimal exogenous progesterone (on the right) if the objective function is $\int_0^{28} \left((P_4(t) - 4)^2 + 0.4u_1 + 1.5u_2^3\right) dt$.

5 Computation time and optimal cost

Here we report computation time and optimal cost for the optimal control simulations.

Table D. Computation time and optimal cost. Computation time (T) and optimal cost (J) for the three therapies. Estrogen monotherapy (Est Mon), Progesterone monotherapy (Prog Mon), and the combination therapy (Comb). All therapies use a time-step $\Delta t = 0.2$. A breakdown of the optimal cost into the three integrals (Int 1, Int 2, and Int 3) comprising the objective function is also presented.

**Treatment**	**Objective function:** $\int_0^{28} \left((P_4(t)-4)^2 + a_1 u_1 + a_2 u_2^4\right) dt$	**T (s)**	**J**	**Int 1**	**Int 2**	**Int 3**
Est Mon	$a_1 = 0.9$; $a_2 = 0$	249067	236.07	171.55	64.52	0
Est Mon	$a_1 = 0.6$; $a_2 = 0$	192656	216.28	171.19	45.09	0
Est Mon	$a_1 = 0.4$; $a_2 = 0$	103044	202.02	170.92	31.10	0
Est Mon	$a_1 = 0.05$; $a_2 = 0$	274871	174.98	170.69	4.29	0
Prog Mon	$a_1 = 0$; $a_2 = 0.16$	31840	105.31	76.39	0	28.92
Prog Mon	$a_1 = 0$; $a_2 = 0.13$	198838	99.67	74.33	0	25.34
Prog Mon	$a_1 = 0$; $a_2 = 0.07$	13498	85.83	64.03	0	21.80
Prog Mon	$a_1 = 0$; $a_2 = 0.005$	59277	22.17	4.92	0	17.25
Comb	$a_1 = 0.4$; $a_2 = 0.7$	344799	137.19	106.59	14.23	16.38

$$\text{where Int } 1 = \int_0^{28} (P_4(t)-4)^2 \, dt, \quad \text{Int } 2 = \int_0^{28} a_1 u_1 \, dt, \quad \text{and} \quad \text{Int } 3 = \int_0^{28} a_2 u_2^4 \, dt.$$

References

[1] Welt CK, McNicholl D, Taylor A, Hall J. Female reproductive aging is marked by decreased secretion of dimeric inhibin. J Clin Endocrinol Metab. 1999;84(1):105-11.

[2] Bormann I. DigitizeIt; 2021. ver. 2.5. Available from: https://www.digitizeit.xyz/.

[3] Margolskee A, Selgrade J. Dynamics and bifurcation of a model for hormonal control of the menstrual cycle with inhibin delay. Math Biosci. 2011;234(2):95-107.
