# Supplementary material for: Toward an optimal contraception dosing strategy
Source: PLoS Comput Biol. 2023 Apr 13;19(4):e1010073. doi: 10.1371/journal.pcbi.1010073 (PMC10101497; doi:10.1371/journal.pcbi.1010073)
Supplement: Attachment — Submitted filename: reviewploscombio2022-1final.pdf [file pcbi.1010073.s002.pdf]

Review of the manuscript entitled "Toward an optimal contraception dosing strategy” for PLOS computational biology

The paper presents a detailed menstrual cycle model which correctly predicts the pituitary and ovarian levels throughout a normal cycle and reflects the effect of exogenous estrogen and progesterone doses. They use this model and optimal control theory to obtain minimum doses for contraception, thereby reducing the side effects of this hormonal therapy. The paper is important and is suitable for the journal, provided the reviewers make the following minor changes.

1. I think the literature survey for applications of optimal control in biology is limited. For example, a recent book entitled “ Control Applications for Biomedical Engineering Systems" academic press(2020) provides several examples, including optimal control related to hormonal treatments. I urge the authors to include all this recent literature.
2. The authors describe the model in Figure 3 but do not provide any information about control variables $u_1(t) = E_2^{exo}(t)$ and $u_2(t) = P_4^{exo}(t)$ in the figure. Please include that so that one can understand the control problem.
3. In the results section, the authors present the residuals for each variable in Table II separately. There is an order of magnitude difference between variables. How did they combine the objective function of residuals (to be minimized) in their analysis? Please provide details.
4. They do not explain how they obtained results for optimal constant dosage. What type of optimization did they use?
5. They talk about computational intensity but do not provide any details. Please provide the details of computational time.
