# Supplementary material for: Toward an optimal contraception dosing strategy
Source: PLoS Comput Biol. 2023 Apr 13;19(4):e1010073. doi: 10.1371/journal.pcbi.1010073 (PMC10101497; doi:10.1371/journal.pcbi.1010073)
Supplement: Attachment — Submitted filename: PCOMPBIOL-D-22-00522_review.pdf [file pcbi.1010073.s003.pdf]

Manuscript No: PCOMPBIOL-D-22-00522

Title: Toward an optimal contraception dosing strategy
Authors: Gavina, Reyes *et al.*

This paper describes the authors' work involving the application of optimal control theory to a modified version of a published model of the menstrual cycle (Margolskee & Selgrade 2011, [11]) in order to establish optimal hormone dosing strategies for effective contraction whilst maintaining progesterone below a critical level. The original model in [11] needed to be modified in order for the contraceptive effects progesterone to be modelled. Their analysis includes optimising constant and time-varying dosing levels of oestrogen and progesterone (individually and combined) over one and multiple cycles. Amongst their conclusions is that the optimal administration of oestrogen is a single dose during the mid follicular phase, whilst progesterone is better administered more-or-less continually. An interesting result is that the disruption of the cycle by oestrogen dose means that the optimal strategy involves non-uniform spacing between doses (at least initially).

This is a well-written, very readable paper in which the authors' undertakes an interesting and potentially useful investigation into optimal control strategies for safe contraceptive dosing. Their approach is very reasonable and as far as I can tell the results are accurate. The only concern I have is the dependence of the results on the choice of terms and parameters within the cost function (see Major comments). If this concern is addressed satisfactorily, then I will have no hesitation on recommending this paper for publication in the journal.

Major comments

It is clear that the observe solutions depend crucially with the choice of cost function and the weighting parameters used. I do not get a sense of how the optimal results depend on the cost function parameters. Furthermore, there are allusions to some challenges in using certain forms of the terms within the function, which are not really discussed in the current draft. For easy reference, the cost function used is

$$\int_{t_0}^{t_f} \left((P_4(t) - P_0)^2 + a_1 u_1 + a_2 u_2^4\right) dt$$

where $u_1 = E_2^{\mathrm{exo}}(t)$ and $u_2 = P_4^{\mathrm{exo}}(t)$. I've made a list of the questions, in no particular order of importance, to help with the authors' response.

(i) Not sure why $(P_4(t) - P_0)^2$ is used, surely there is no cost in the context of safe dosing for $P_4 < P_0$. A sentence clarifying this issue is needed.

(ii) I don't understand "These weights are selected using an iterative process to produce desired P_4 levels". The values of $a_1 = 0.4\,\mathrm{pg}^2/\mathrm{mL}^2$ and $a_2 = 0.7\,\mathrm{mL}^2/\mathrm{ng}^2$ seem to be prescribed rather than found through some iterative process. Furthermore, if such an iterative process was implemented, what is the convergence criteria for the iterative process?

(iii) To illustrate the effect of parameters a_1 and a_2 on the optimal solutions, it would be good to have for the single hormone, constant dosing cases plots of the optimal u_1 against a_1 and u_2 against a_2; this should help the reader understand better the role of a_1 and a_2 on the optimal solutions.

(iv) Putting together points (ii) and (iii), more explanation is needed on the choice of a_1 and a_2 in their optmisations.

(v) The authors' state "Very oscillatory results are obtained for power of u_2 less than four". Is this saying that the "optimum" u_2 is oscillatory or is this in terms of convergence behaviour? Their solution to the problem seems a little arbitrary and it appears odd to me that the form of the cost terms for u_1 and u_2 are different. The discussion on this needs expanding.

(vi) There seems to be a units mismatch in the function. The units of $(P_4(t) - P_0)^2$ is presumably $\mathrm{ng}^2/\mathrm{mL}^2$, but $a_1 u_1$ seems to be $\mathrm{pg}^2/\mathrm{mL}^2$. This needs fixing or explaining.

(vii) In their optimum solutions, I do not have a sense whether the greatest cost in an optimised solution comes from the $\int_{t_0}^{t_f} (P_4(t) - P_0)^2 \, dt$ term, the other terms or that they are all about the same size. A brief "We found that ..." type statement on page 15 would be good to address this.

Minor comments

1. Least squares formulation on Page 14. Why multiply the relative errors by a 100? This effectively multiplies the weighting terms by 10 and surely has no impact on the results.

2. Figure 14 is very interesting. Close inspection of P_4 in Fig 14B suggests the optimal dosing solutions have period-doubled, with maximal P_4 peaks occurring on alternate cycles. Furthermore, it appears from fig. 14B that the optimal oestrogen doses are becoming equally spaced apart - is this correct? This should be mentioned in the discussion, since such dosing strategy would be much easier to prescribe for the user.

Typos

Couldn't find any!
