# Supplementary material for: Toward an optimal contraception dosing strategy
Source: PLoS Comput Biol. 2023 Apr 13;19(4):e1010073. doi: 10.1371/journal.pcbi.1010073 (PMC10101497; doi:10.1371/journal.pcbi.1010073)
Supplement: Attachment — Submitted filename: PLoSCompBiol_response.pdf [file pcbi.1010073.s004.pdf]

Toward an optimal contraception dosing strategy

Brenda Lyn A. Gavina, Aurelio A. de los Reyes V, Mette S. Olufsen,
Suzanne Lenhart, Johnny T. Ottesen

RESPONSE TO REVIEWERS' COMMENTS

November 1, 2022

REFEREE 1

We thank the reviewer for carefully reading our manuscript providing constructive suggestions for edits. We have addressed all your comments below indicating changes that we have made to the manuscript in blue.

Major comments

- The authors used to fit their multi-parametric model the Nelder-mead algorithm using a least squares error criteria. The dataset actually used is not available to the reader making not totally clear what is meant by the repetition of the data four times and what z-scores are used.

  Response: Data used to calibrate the model are only measured over one cycle (28 days). These data, extracted from Figure 1 in [1] using the software DigitizeIt version 2.5 [2], are shown in Supplementary A. Assuming that these data represent periodic steady state we repeated data to demonstrate that the model has a periodic solution. This is illustrated in Figure 4. This figure shows periodic model predictions for four cycles. For each cycle the model predictions are shown together with the data, i.e., the data is "repeated" four times. To show how well the model fits to data we have calculated the sum of squared residuals and displayed that with the data in Table II. The residuals were computed over the last cycle. We have changed discussion of model calibration (page 14) as well as Figure 4 and figure caption for this figure.

  Methods (Page 5, line 158):
  The output of the mathematical model without the administration of exogenous hormone is compared to the 28-day data (solid circles in Figure 2) from Welt et al. [1]. These data (see Supplementary A), extracted from Figure 1 in [1] using the software DigitizeIt version 2.5 [2], comprise mean levels of E_2, P_4, $Inh\ A$, LH, and FSH taken from 23 normally cycling younger women aged 20 to 34 years.

  Methods (Page 9, lines 261–271):
  The weight $w_{H^*}(i)$ equals 1 except for:

  i. $w_{E_2^*}(13) = 1.58 =$ z-score of 13th E_2 data (the maximum E_2 data),
  ii. $w_{P_4^*}(21) = 1.39 =$ z-score of 21th P_4 data (the maximum P_4 data),
  iii. $w_{Inh^*}(20) = 1.35 =$ z-score of 20th Inh data (the maximum Inh data),
  iv. $w_{LH^*}(14) = 2.16 =$ z-score of 14th LH data (the maximum LH data), and
  v. $w_{FSH^*}(14) = 1.80 =$ z-score of 14th FSH data (the maximum FSH data).

M is the number of data points and N is the combined number of model parameters and initial conditions. The M data points is composed of four repetitions of the 28-day Welt data as shown in Figure 4. The difference $M - N$ in the denominator ascertains that errors don't increase with repetition of the data set.

Supplementary A (Table 1):

Supplementary A: Data and standard deviation of model parameters

Table : Data extracted from Figure 1 in [1] using the software DigitizeIt version 2.5 [2], comprise mean levels of E_2, P_4, $InhA$, LH, and FSH taken from 23 normally cycling women.

**Day**	$\mathbf{E_2}$ [pg/mL]	$\mathbf{P_4}$ [ng/mL]	**InhA** [IU/mL]	**LH** [IU/L]	**FSH** [IU/L]
1	51	1.1	1.0	12	11.4
2	55	0.6	1.1	14	11.6
3	53	0.6	1.0	15	11.7
4	59	0.6	1.1	14	12.4
5	60	0.6	1.1	17	12.6
6	62	0.6	1.0	17	11.3
7	66	0.6	1.1	19	12.1
8	72	0.6	1.2	18	11.3
9	95	0.6	1.7	17	10.0
10	119	0.6	2.3	17	8.7
11	138	0.6	3.2	17	8.6
12	188	0.6	4.5	25	8.2
13	237	0.7	7.4	50	10.4
14	215	1.2	9.3	123	19.6
15	127	2.0	7.7	41	12.1
16	91	5.0	8.1	22	9.2
17	102	8.9	10.1	20	8.7
18	119	11.2	8.9	20	8.6
19	140	15.6	9.5	18	7.4
20	133	17.3	11.5	16	7.2
21	152	17.9	9.1	12	6.1
22	142	17.2	8.7	9	5.4
23	140	14.4	7.5	11	5.2
24	155	12.6	6.6	10	5.4
25	133	10.3	5.7	11	5.3
26	114	8.1	4.1	11	6.1
27	70	4.3	2.2	11	6.7
28	55	1.9	1.7	11	8.3

Results (Figure 4):

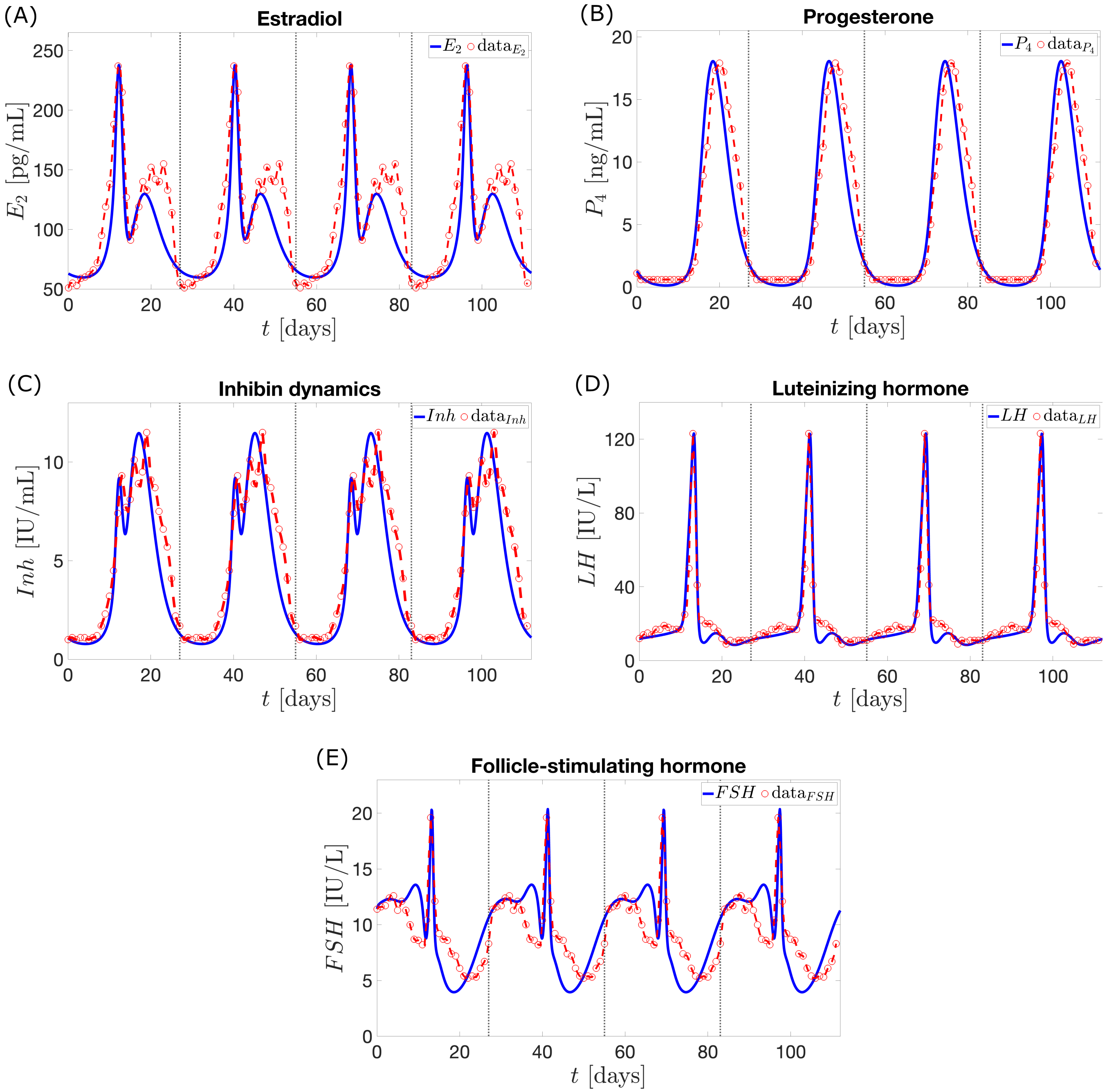

Figure : **Normal cycle solution.** The blue curves describe the dynamics of the pituitary and ovarian hormones predicted by the model without exogenous estrogen and progesterone. The vertical lines partition the red curves into four cycles. Each partition presents the 28-day normal cycle hormone data extracted from Welt et al. [1]. The model output, which has period 28.05 days, gives a good fit as shown in Table III.

- As the Nelder-Mead algorithm can be very slow, they do not mention if they experienced convergence issues. In addition no information is given about the variance of the estimated parameters.

  Response: The reviewer is correct, that the Nelder-Mead algorithm can be slow to converge. Our simulation with fminsearch though gives an exitflag of 1, indicating that the algorithm converged to a solution [3]. To explore the variance of the estimated parameters, we applied bootstrapping with 1000 random initial guesses and presented the result in Supplementary A.

Supplementary A (Table 2):

Supplementary A: Data and standard deviation of model parameters

Table : **Standard deviation of model parameters (Par) and initial conditions (IC).** Mean and standard deviation of parameters and initial conditions obtained from bootstrapping with 1000 random initial guesses.

**Par/IC**	**Value**	**Unit**	**Mean ± Standard deviation**
k_{LH}	0.9661	day^{-1}	1.0212 ± 0.2854
$V_{0,LH}$	550.03	IUday^{-1}	627.08 ± 28.44
$V_{1,LH}$	3329.19	IUday^{-1}	3499.11 ± 841.25
km_{LH}	136.05	pgmL^{-1}	164.52 ± 16.28
$ki_{LH,P}$	6.78	ngmL^{-1}	7.55 ± 2.01
$c_{LH,E}$	0.0060	mLpg^{-1}	0.0053 ± 0.0025
$c_{LH,P}$	1.98	mLng^{-1}	2.11 ± 0.83
V_{FSH}	294.90	IUday^{-1}	274.55 ± 16.36
k_{FSH}	14.59	day^{-1}	14.95 ± 8.88
$c_{FSH,E}$	0.0151	$\left(\frac{mL}{pg}\right)^2$	0.0147 ± 0.0048
$Ki_{FSH,Inh}$	16.83	IUmL^{-1}	17.79 ± 7.26
$c_{FSH,P}$	52.31	mLng^{-1}	44.89 ± 24.26
b	0.0453	$\text{L}\mu\text{g(IUday)}^{-1}$	0.0550 ± 0.0227
c_1	0.1036	$\text{LIU}^{-1}\text{day}^{-1}$	0.1230 ± 0.0202
c_2	0.0577	$(\text{L/IU})^{\alpha}/\text{day}$	0.0502 ± 0.0185
c_3	0.0170	L/(IUday)	0.0142 ± 0.0053
c_4	1.14	$(\text{L/IU})^{\gamma}/\text{day}$	0.91 ± 0.36
d_1	0.7537	day^{-1}	0.7758 ± 0.1288
d_2	0.6866	day^{-1}	0.7558 ± 0.1225
k_1	0.6699	day^{-1}	0.7393 ± 0.1153
k_2	0.6388	day^{-1}	0.6503 ± 0.0960
k_3	0.9191	day^{-1}	0.7092 ± 0.1237
k_4	1.88	day^{-1}	1.59 ± 0.34
α	0.9505		1.0163 ± 0.1320
γ	0.1615		0.2337 ± 0.1634
e_0	57.60	pg/mL	52.24 ± 3.77
e_1	0.0269	L^{-1}	0.0638 ± 0.0433
e_2	0.4196	L^{-1}	0.4265 ± 0.1468
e_3	0.4923	L^{-1}	0.5991 ± 0.1310
p_1	0.0032	kL^{-1}	0.0025 ± 0.0013
p_2	0.1188	kL^{-1}	0.0964 ± 0.0225
h_0	0.6606	IUmL^{-1}	0.7096 ± 0.0958
h_1	0.0193	$\text{IUmL}^{-1}\mu\text{g}^{-1}$	0.0246 ± 0.0069
h_2	0.0159	$\text{IUmL}^{-1}\mu\text{g}^{-1}$	0.0147 ± 0.0052
h_3	0.0119	$\text{IUmL}^{-1}\mu\text{g}^{-1}$	0.0163 ± 0.0077
w	9.21	ng/mL	11.87 ± 3.64
q	5.11	ng/mL	6.06 ± 2.06
$RP_{LH}(0)$	167.57	IU	155.52 ± 66.54
$LH(0)$	11.81	IU/L	11.02 ± 3.01
$RP_{FSH}(0)$	14.48	IU	13.41 ± 11.87
$FSH(0)$	11.41	IU/L	9.86 ± 3.45
$RcF(0)$	2.10	ng	2.18 ± 0.81
$GrF(0)$	4.12	ng	5.23 ± 5.78
$DomF(0)$	0.46	ng	0.48 ± 0.61
$Ov_1(0)$	1.06	ng	1.19 ± 1.46
$Ov_2(0)$	1.67	ng	2.26 ± 4.12
$Lut_1(0)$	4.16	ng	6.28 ± 7.59
$Lut_2(0)$	13.03	ng	18.44 ± 11.58
$Lut_3(0)$	16.48	ng	14.79 ± 5.93
$Lut_4(0)$	10.29	ng	9.54 ± 2.69

- The objectives of minimizing the exogenous exposure to both estrogen and progestin over time in order to keep progesterone below a fixed threshold are clearly stated. Some additional explanations could have been added regarding the influence of the second and third terms in the integrand as well as the choice of the P_0 value. Is the delay tau fixed or optimized in the process and how it is either fixed or initialized?

  Response: We considered the biological reason provided by Margolskee et al. [4] for using 1.5 days as Inhibin delay so we kept this value and not included it in the parameters we optimized. It is a constant in the mathematical model we optimized.
  The objective function $\int_0^{28} \left((P_4(t) - 4)^2 + a_1 u_1 + a_2 u_2^4\right) dt$ is decomposed to its components $\int_0^{28} \left((P_4(t) - 4)^2\right) dt$, $\int_0^{28} (a_1 u_1)\, dt$, and $\int_0^{28} (a_2 u_2)\, dt$ (see Supplementary E) to investigate more the influence of the second and third terms in the integrand. This shows which component contributes more to the cost and in turn, provide guidance on how the weights can be adjusted to attain desired P_4 level.
  The objective of this study is to determine the minimum total dosage of exogenous hormone that will prevent ovulation. Simulations show that lowering P_4 level further requires enlarging the dosage. Therefore, we sought a target level below but close to 5 ng/mL ensuring a small dose of exogenous hormones, i.e., we are maximizing $P_4(t) < 5$ ng/mL. This objective was behind the term $\left((P_4(t) - P_0)^2\right)$ in the objective function which is standard for getting $P_4(t)$ to track the target P_0, to minimize deviation of $P_4(t)$ from P_0 [5]. Simulations start with $P_4(t)$ below P_0 as we aim to get near P_0. We tried different target points starting with 5 ng/mL, and P_0 equal to 4 ng/mL does a good job by allowing easier manipulation of weights. For instance, if $P_0 = 4$ ng/mL, the values of the weights a1 and/or a2 do not have to be very low to make P_4 concentration less than 5 ng/mL. To make this clearer we have revised the description of the term $\left((P_4(t) - P_0)^2\right)$ on Page 15.
  We have revised the Methods section as follows:

  Methods (Page 10, lines 293–297): The term $\left((P_4(t) - P_0)^2\right)$ in the integrand is standard for getting $P_4(t)$ to track the target P_0 [5]. Because we want to keep $P_4(t) < 5$ ng/mL for $0 = t_0 < t_f = 28$, a target point P_0 must be lower than 5 ng/mL. In addition, simulations for constant dosage show that lowering P_4 level further requires higher dose of exogenous hormones. Thus, we take $P_0 = 4$ ng/mL, a value lower than but close to 5 ng/mL as target point.
  Methods (Pages 10–11, lines 312–314):
  The constants $a_1 > 0$ an $a_2 > 0$ are weight factors which balance the effort of minimizing u_1 and u_2, and hitting P_0. We found that low values of weights produce small value of $\int_{t_0}^{t_f} (P_4(t) - P_0)^2\, dt$ (see Supplementary E), i.e., $P_4(t)$ is near P_0, but increases exogenous hormone dose.

- The reproductive function in women is a very complex multiscale dynamical system highly exposed to both endogenous and exogenous factors. It explains both the large intra- and between women variability of the menstrual cycle length over the reproductive period.

  The reviewer was very concerned by the total absence of discussion of this aspect as the only dataset used by the authors concern daily mean values of measured blood concentrations of pooled menstrual cycles in normally cycling women. The dataset was extracted from a figure from a single article (Welt et al.). No information is given how hormonal profiles from different women were scaled together. Was it scaled according to their first day of their respective cycles or a posteriori according to the day of the putative ovulation (LH peak)? Hence, the model is adjusted to an ideal profile which does not correspond to any existing woman. The model would have been more convincing if fitted to the individual hormonal profiles.

  Response: As stated in the methods section (Page 5), the data used in this study, extracted from Welt et al. [1] reports average values from 23 normally cycling women. The data were centered to ovulation based on three of the following days: i) LH peak, ii) midcycle FSH peak iii) on or after the midcycle E2 peak, and iv) doubling of P_4 from baseline or P_4 level getting 0.6 ng/mL [1]. Before

studying contraception, the model was calibrated to these data. And the calibrated model was used in the optimal control simulations.

We agree with the reviewer, it would be beneficial to show results for more data. We have commented on this limitation in the Discussion (page XX).

Methods (Page 11, lines 335–346): This study suggests a method/tool on how ovulation can be suppressed for an example woman with a specific cycle length. Though we are presenting a patient-specific and not a population study, the method we described can be repeated to any woman if her hormone levels are known. To illustrate the adaptability of our method to women with different cycle lengths and understand how predictions change with variation in the period, we performed the following:

i. sensitivity analysis (shown in Supplementary B) to determine the parameters which greatly affect the model cycle length, then

ii. from the most sensitive parameters, we took some and vary them to make various cycle lengths, and

iii. applied the optimization code to determine timing of administration and dosage of exogenous hormones that result in anovulation.

Conclusion (Page 19, lines 617–625):
Our work suggests a method of obtaining an optimal regimen for administration of exogenous hormone/s over one cycle. In the future when more cost-effective optimization scheme is accessible, one may build on this process to investigate dosing schemes for multiple cycles. Currently, the process applied over one cycle may be repeated for every cycle. It is also possible to use the result over one cycle to continually suppress ovulation over multiple cycles. Additionally, because there is a biomarker for level of hormones like E2, P4, and LH, the results presented here give insights on construction of timed devices that give contraception at certain parts of the menstrual cycle.

Supplementary B (Figure 1):

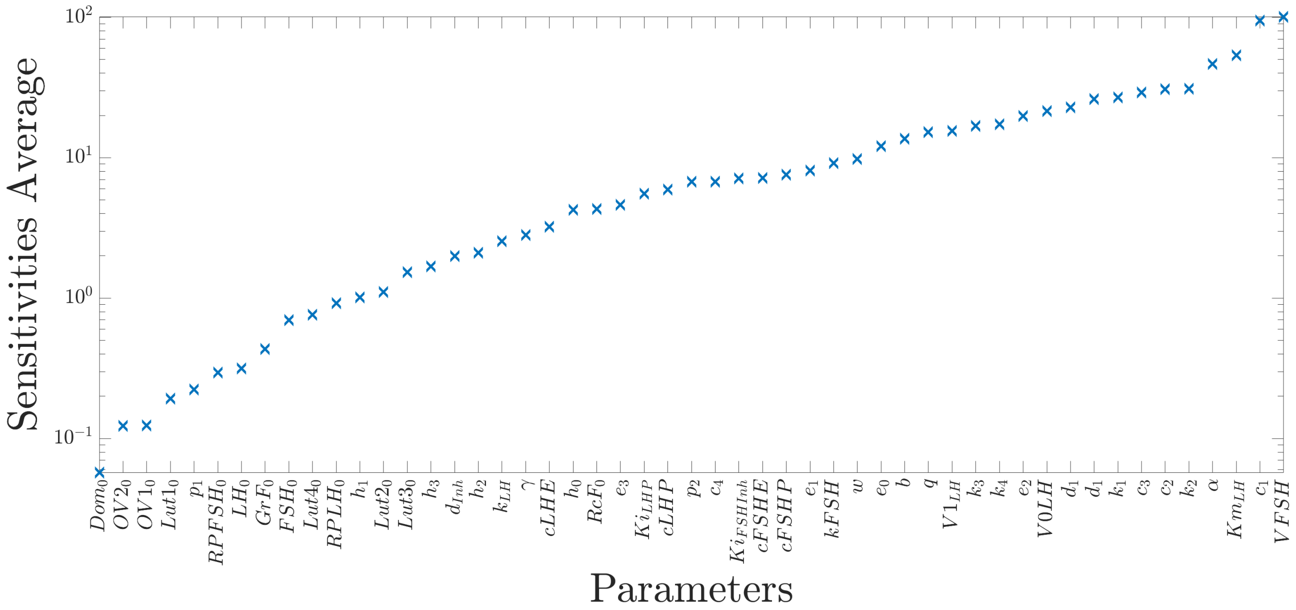

Figure : **Sensitive parameters.** Sensitivity analysis reveals parameters that affect the model output most.

Discussion (Page 18, lines 577–588)

Result of the sensitivity analysis performed on the model (see Supplementary B) shows parameters which largely affect model output. To explore adaptability of our control method to different menstrual cycle conditions, we perturb some of the most sensitive parameters to generate model output with different cycle lengths and peaks. Because of its biological significance, we perturbed the third most sensitive parameter km_{LH}. This parameter is the E_2 value at half saturation, it signals strong stimulation in the production of E_2 necessary for ovulation. With km_{LH} = 115 pg/mL and 160 pg/mL, we yield model outputs of cycle length 26.92 days and 29.08 days, respectively. Applying the code for optimal non-constant estrogen monotherapy gives the optimal control u_1 profile in Figure 15B. The effect of the control in Figure 15B on the P_4 level is illustrated in Figure 15C. These figures show how the optimal control dosage and timing of administration adjust to the varied cycle types.

Discussion (Figure 14):

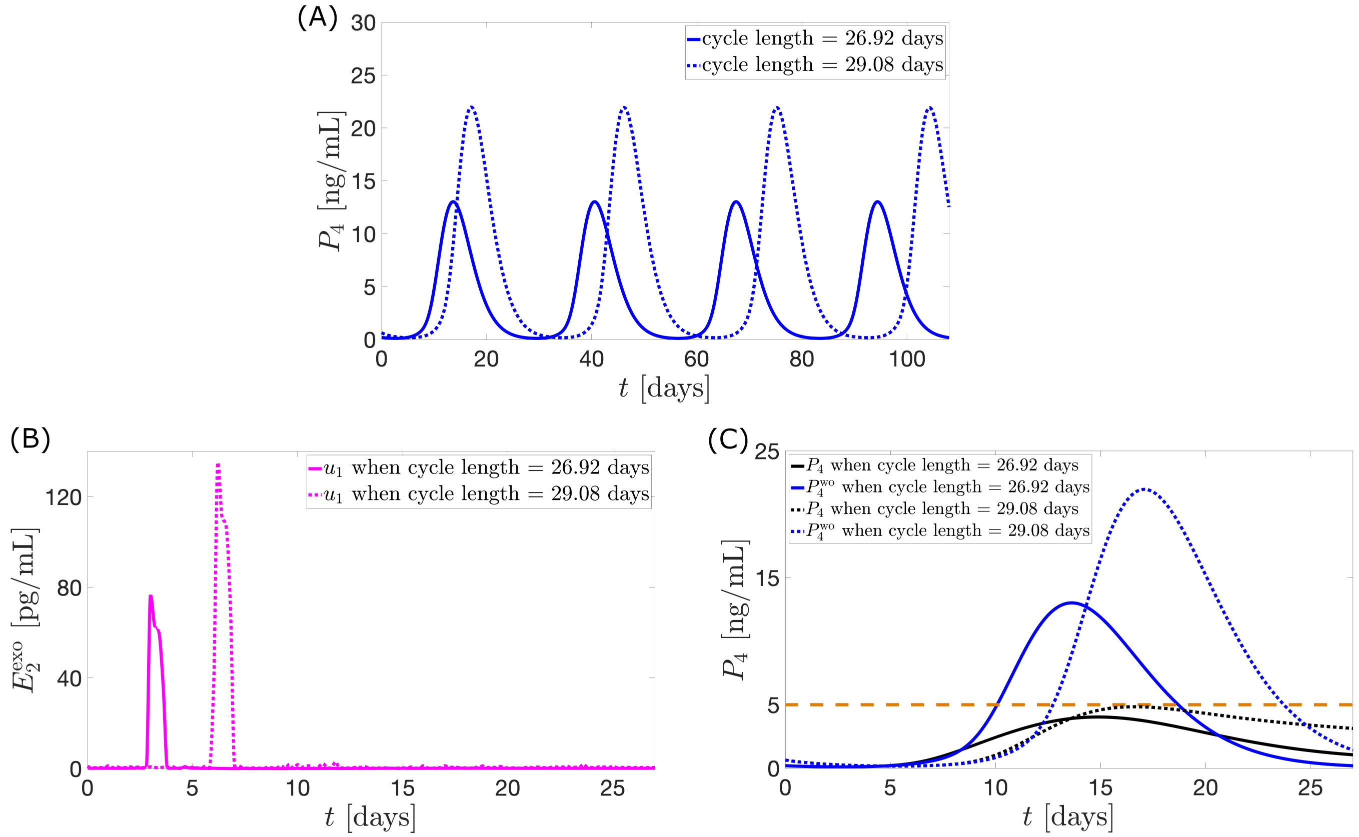

Figure : **Optimal control u_1 on different cycle lengths.**

- The goal of any contraceptive method, whatever is the method, is to insure a protection against an unwanted pregnancy over a period of time, not restricted to a single menstrual cycle. It is classically measured using the Pearl index corresponding to the number of failures over a two year period i.e. 24000 women x cycles assuming menstrual cycle of an ideal one month duration. The present mathematical approach does not address this very important issue. Furthermore, several strong assumptions are made which limit the conclusions of the authors.

  Response: Clinically it is true that contraception requires the method to work over a period of time. The results presented here is meant as a proof of concept understanding how to disrupt the system to make cycling stop. The objective of this study is to show that by impacting just a few days it is possible to stop cycling. The control could be repeated for many month achieving non-cycling for a prolonged period of time. To make this clear, we have added text to the Introduction and Discussion addressing this point

  Introduction (Page 3, lines 88–89)
  It is a patient-specific study that describes a methodology of blocking ovulation.

  Discussion (Pages 17–18, lines 539–560):
  For instance, if the large-dose portion of u_1 is applied in equal intervals from day 35 to day 280 (see Figure 15, anovulation is no longer induced beginning on the 5th 28-day period (i.e. from day 140 to day 280). This is because the application of u_1 in the preceding period changes the dynamics of the menstrual cycle in the succeeding days. One of these changes is the cycle length. Now, because the control u_1 must be administered at a time before E_2 surge (when u_1 is not applied), the timing of administration in the next cycles must also be changed to continuously suppress ovulation. Sometimes resulting cycle length is less than 28 days so only a portion of u_1 will be applied. In

addition, because lower levels of u_1 are negligible compared to the higher levels, we applied only the large-dose portion of u_1 from day 35 to day 280 (see Figure 15). The administration is done when E_2 level is increasing and reaches 75 pg/mL. We are currently conducting further investigation to determine the highest E_2 hormone level where administration of u_1 would commence to still inhibit ovulation. Note that although u_1 is applied eighteen times (in unequal intervals) from day 0 to day 280, the total dosage is still significantly lower than the total constant dosage of exogenous E_2 which induces anovulation. The dosing regimen presented, where administration is triggered by a specific biomarker, here the 75 pg/mL-E_2 level, offers insights on construction of timed devices that give contraception at certain parts of the menstrual cycle. For instance, contraceptive dose may be given relative to E_2 level. So even if some patients' cycle length vary a little bit from month to month, their E_2 level can be measured to know when to make a contraceptive device spike.

Discussion (Figure 15):

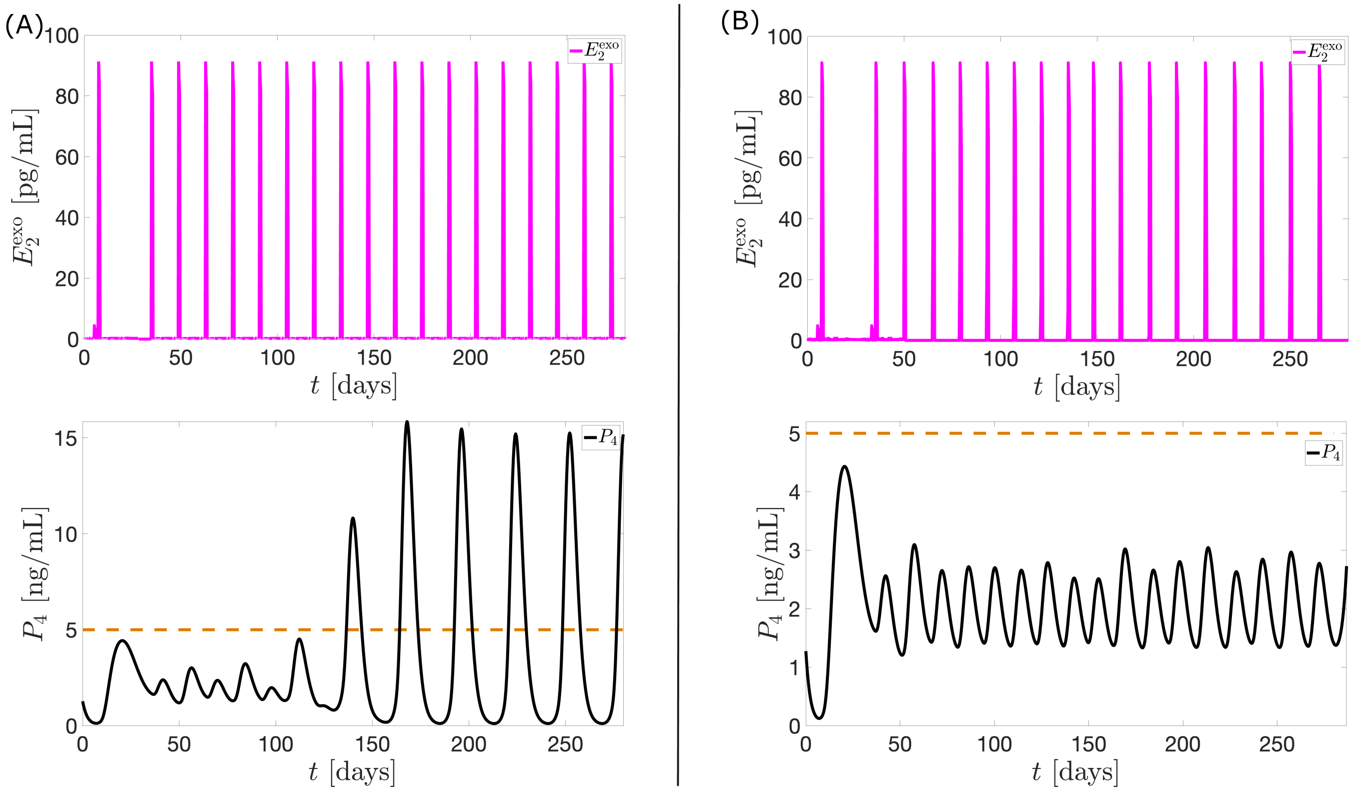

Figure : Multiple application of optimal control u_1. The black curve is the model output when multiple u_1 (magenta curve) is applied. In panel (A), the application of u_1 from day 35 to day 280 in equal intervals is unable to sustain anovulation. Panel (B) shows a scheme for administration of multiple u_1 which continuously blocks ovulation.

We will address the assumptions one at the time:

Assumption 1: page 11, last paragraph, the statement appears in contradiction with the corresponding sentence (*the clearance of the ovarian hormones is faster than the clearance of pituitary hormones*). Indeed the clearance of steroids is in the range of hours as compared to the one of peptide hormones which is in the range of minutes.

Response: In the original manuscript submitted, the last paragraph of page 11 states "*Exogenous estrogen and/or progesterone inhibit ovulation through different mechanisms. Estrogen causes anovu-*

lation by suppressing gonadotropin secretion, depicted in the release term of Equations (1) and (3)." We are not sure if this is the statement that contradicts the assumption on the difference between clearance of the ovarian and pituitary hormones.

Assumption 2: during a menstrual cycle, the FSH level starts rising earlier than it is assumed in the article. It starts rising indeed at the end of the previous luteal phase and the early days of menstruation, hence allowing the growth recovery of the follicles reacting to FSH.

Failure to restart the hormonal contraceptive in time, whatever is its composition, allows the normal physiological process of selection and dominance ending up with ovulation to resume. Shortening the period and the level of exogenous hormone exposure to avoid ovulation as suggested by the authors according to their simulations, mirrors the methods and apps proposed for the natural control ovulation, the reliability of those always assuming normally cycling women in a stable state. It contradicts the principle of a safe contraceptive means open to all women from all conditions, environmental exposures and ages.

Response: The authors agree with the reviewer's comment that the model was not able to incorporate certain biological aspects in great detail and that the model has been calibrated to normally cycling women. However, the modeling framework could be adaptable to women-to-women and within-women menstruation variability. Due to insufficient access to real measurements, we created a synthetic data that captures different menstrual cycle length and hormonal levels as discussed previously. We show the plots for these once again.

Discussion (Figures 14–15):

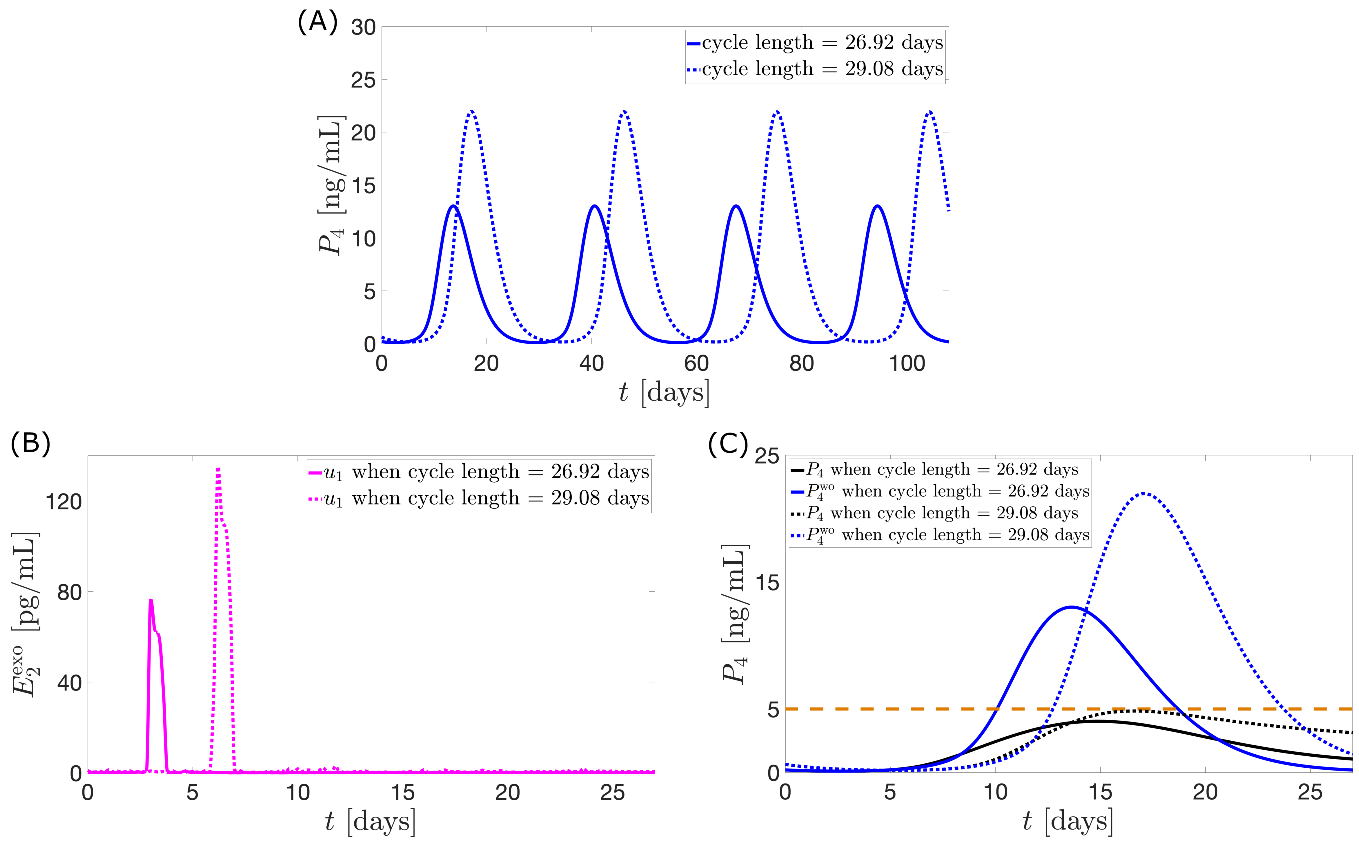

Figure : **Optimal control u_1 on different cycle lengths.**

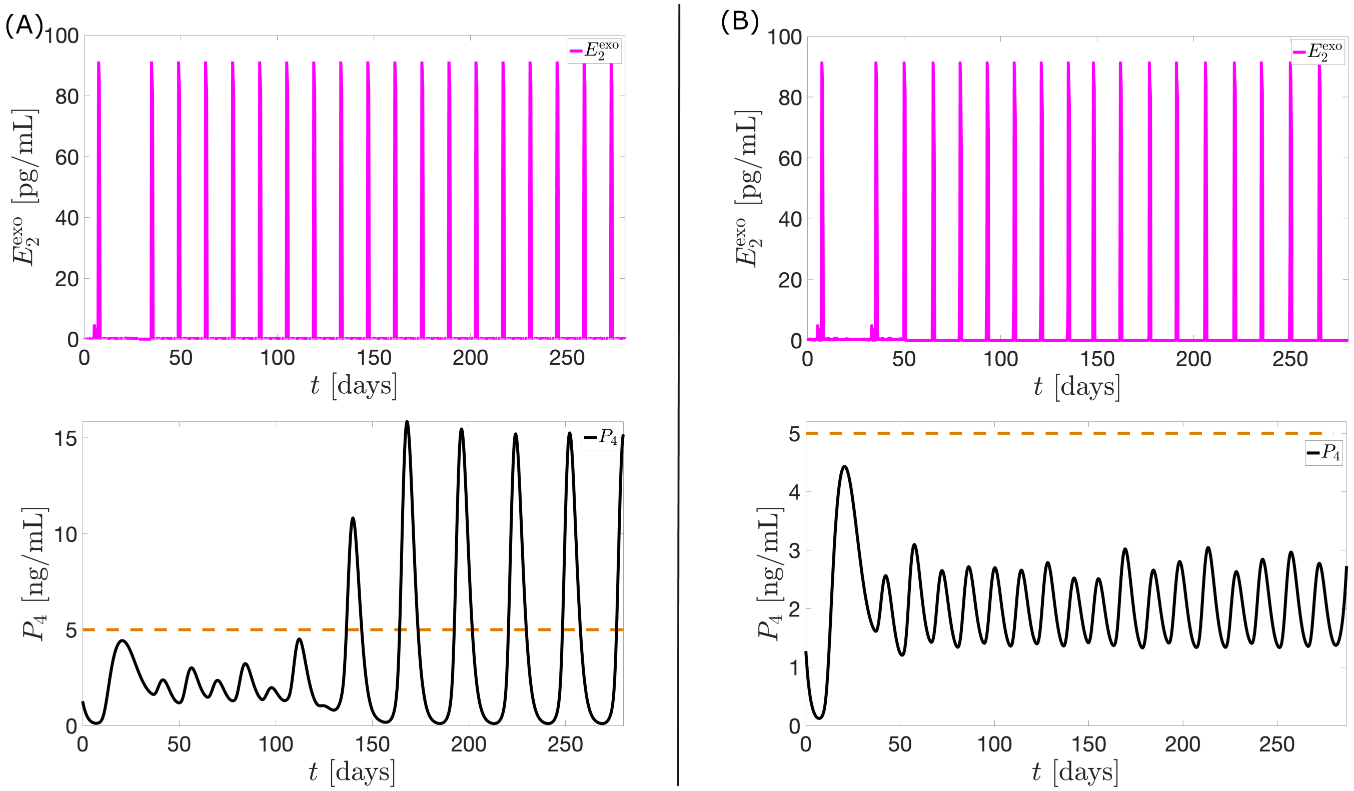

Figure : Multiple application of optimal control u_1. The black curve is the model output when multiple u_1 (magenta curve) is applied. In panel (A), the application of u_1 from day 35 to day 280 in equal intervals is unable to sustain anovulation. Panel (B) shows a scheme for administration of multiple u_1 which continuously blocks ovulation.

Assumption 3: A precise menstrual timing is known in all women. Several studies have estimated the probability of conception according to the day of the menstrual cycle. Of course conception is not superimposable to ovulation but it means at least that an ovulation occurred right before the conception. The probability of ovulation starts rising as early as the 6th day of the cycle and remains elevated until late in the cycle corresponding to ovulation disorders.

Ferreira-Poblete A. Adv Contracept. 1997; 13:83-95. The probability of conception on different days of the cycle with respect to ovulation: an overview.

Royston P. Stat Med. 1991; 10:221-40. Identifying the fertile phase of the human menstrual cycle.

Lynch C et al. Paediatr Perinat Epidemiol. 2006; 20 Suppl 1:3-12. Estimation of teh day-specific probabilities of conception: current state of the knowledge and the relevance for epidemiological research.

Stirnemann JS et al. Hum Reproduct. 2013; 28:110-116. Day-specific probabilities of conception in fertile cycles resulting in spontaneous pregnancies.

All these studies strongly suggest at least an absence of a precise knowledge of the menstrual timing in a large fraction of women when initiating a hormonal contraception. It explains why the time for initiating oral contraception should be very early in the menstrual cycle in order to block the ovulation. The following <<pill cycles>> are indeed artificial and cannot be interpreted as menstrual cycles. The exposure to exogenous hormones induces an artificial regulation of the reproductive axis with differences from one woman to another.

Response: Indeed the model is limited to having prior knowledge on the menstrual timing to effectively initiate a hormonal contraception and could be integrated in further modeling iteration subject to availability of patient data and specifics of the hormonal contraceptives. We have included this in the limitation of the study.

Discussion (Page 19, lines 589–594):
Though the methodology utilized in this study can be adapted to varied cycle conditions as discussed, we recognize some limitations. For instance, several studies have reported that menstrual timing is not precisely known in all women [6, 7, 8, 9]. A prior knowledge of the menstrual timing can effectively identify appropriate administration schedule of hormonal contraception. Thus, further investigation and modeling iteration is needed in this direction.

Assumption 4: An important assumption is made regarding the effect of exogenous steroids supposed to have the same effect as endogenous natural hormones. Actually, exogenous hormones used in contraceptives do not act totally as endogenous hormones. This is clear for progestins whose effects are different regarding both their metabolisms and their effects. Natural progesterone has strong physiological effects which differ from synthetic progestin effects used in hormonal contraceptives. The effects of the latter differ according to their nature and their androgenic effects, with an impact of GnRH pulsatility and their capacity to inhibit ovulation when used alone. Natural progesterone is available as a drug and frequently prescribed in reproductive medicine either orally or vaginally but its metabolism presents a large variability which does not allow its use for ovarian blockade.

Similarly, giving progesterone receptor modulator compounds including progestins during the follicular phase of the menstrual cycle will affect the normal course of the cycle either by suppressing or simply delaying ovulation depending of the follicular status. It remains a serious matter of concern when trying to estimate the failure of emergency hormonal contraception as many of the few unwanted pregnancies observed in this situation can be attributed to delayed ovulations after exposure to high levels of steroid hormones.

Similar observations in terms of metabolism can be made about ethinyl estradiol whih represents the more frequent estrogen component in hormonal oral contraceptives.

Hormonal contraceptives using natural estradiol have been recently proposed with similar limitations.

Experiments in Rhesus monkeys have clearly shown that estradiol given in the mid or late follicular phase shuts down the GnRH pulsatility hence inhibiting ovulation (see for instance Grosser et al. Neuroendocrinology. 1993 Jan;57(1):115-9; O'Byrne K et al. Neuroendocrinology. 1993 Apr;57(4):588-92.), though a similar effect has been debated in humans.

Response: The authors agree with the reviewer that in the current study, exogenous steroids are assumed to have a similar effect as the endogenous natural hormones and obviously a clear limitation. This has been pointed out to be an important consideration for further modeling studies.

Conclusion (Page 19, lines 611–613): Additionally, the mathematical model can be modified to capture the distinction between exogenous and endogenous hormones, allowing investigation into effects of specific drugs.

- Reproducibility of the results: Except from the fact that the dataset extracted from a figure published in the article by Welt et al. is not directly provided, the reproducibility of the mathematical part appears good. It is not clear what is behind the <<manual adjustments>> carried out after optimization. No direct code is provided.

Response: We have included the dataset extracted from Welt et al. [1] in Supplementary A.
We ran the Matlab function fminsearch to estimate 37 model parameters and 13 initial conditions. This is a 50-dimensional space where we don't know the details of the landscape. The algorithm investigates that partly but only locally so we can't guarantee that the algorithm would go around a hump to find the best minima, we only get a local one. When the algorithm ended, it gave the exitflag 1 showing convergence [3]. Our model has to oscillate on its own periodically so it's much harder than any optimization problem where we are not getting cycling from the data hence, it can be tricky to do the optimization. In addition, we are fitting our model to the Welt data set which has peaks on it, making optimizers easily miss these details. Therefore, we had to apply manual tuning to make the model output cycle length close to the data cycle length of 28 days and attain the data peaks. In the future, more work could be put in to employ more advanced optimization algorithms in estimating the parameters. For example, one may consider the basin approach.
We have included here a link to some of the codes.

https://github.com/3r3nd/menstrual-cycle-project

- Substantial evidence for the proposed conclusions. In its present form, the article describes an interesting mathematical exercise based n a series of strong assumptions and a rather limited dataset corresponding to daily mean hormonal values of normally cycling women. All together, it strongly limits the possibility to translate its results to real contraceptive issues.

  Response: The reviewer is right that the results of the study is not directly translatable to real patients but the principles proposed here could easily be translated. As noted earlier, the objective of this study is to show that optimal control can be used to identify when to give contraceptive treatment. This manuscript is aimed at describing a methodology, not conducting analysis of a large dataset. As discussed in earlier responses we have clarified the mission of this study in the Introduction and in more detail discussed its limitations. We hope that the method we presented can be generalized so that it can be used on a lot of different patients provided patient data and specifics of the drug are known. If we know more specific information about a contraceptive, details can be added to the mathematical model allowing better predictions.

REFEREE 2

We thank the reviewer for carefully reading our manuscript providing constructive suggestions for edits. We have addressed all your comments below indicating changes that we have made to the manuscript in blue.

Major comments

It is clear that the observed solutions depend crucially with the choice of cost function and the weighting parameters used. I do not get a sense of how the optimal results depend on the cost function parameters. Furthermore, there are allusions to some challenges in using certain forms of the terms within the function, which are not really discussed in the current draft. For easy reference, the cost function used is

$$\int_{t_0}^{t_f} \left((P_4(t) - P_0)^2 + a_1 u_1 + a_2 u_2^4 \right) dt,$$

where $u_1 = E_2^{\text{exo}}(t)$ and $u_2 = P_4^{\text{exo}}(t)$. I've made a list of the questions, in no particular order of importance, to help with the authors' response.

(i) Not sure why $(P_4(t) - P_0)^2$ is used, surely there is no cost in the context of safe dosing for $P_4 < P_0$. A sentence clarifying this issue is needed.

Response: The objective of this study is to determine the minimum total dosage of exogenous hormone that will prevent ovulation. Simulations show that lowering P_4 level further requires enlarging the dosage. Therefore, we sought a target level below but close to 5 ng/mL ensuring a small dose of exogenous hormones, i.e., we are maximizing $P_4(t) < 5$ ng/mL. This objective was behind the term $\left((P_4(t) - P_0)^2\right)$ in the objective function which is standard for getting $P_4(t)$ to track the target P_0, to minimize deviation of $P_4(t)$ from P_0 [5]. Simulations start with $P_4(t)$ below P_0 as we aim to get near P_0. We tried different target points starting with 5 ng/mL, and P_0 equal to 4 ng/mL does a good job by allowing easier manipulation of weights. For instance, if $P_0 = 4$ ng/mL, the values of the weights a1 and/or a2 do not have to be very low to make P_4 concentration less than 5 ng/mL. To make this clearer we have revised the description of the term $\left((P_4(t) - P_0)^2\right)$ on Page 15.

Methods (Page 10, lines 293–297): The term $\left((P_4(t) - P_0)^2\right)$ in the integrand is standard for getting $P_4(t)$ to track the target P_0 [5]. Because we want to keep $P_4(t) < 5$ ng/mL for $0 = t_0 < t_f = 28$, a target point P_0 must be lower than 5 ng/mL. In addition, simulations for constant dosage show that lowering P_4 level further requires higher dose of exogenous hormones. Thus, we take $P_0 = 4$ ng/mL, a value lower than but close to 5 ng/mL as target point.

(ii) I don't understand "These weights are selected using an iterative process to produce desired P_4 levels". The values of $a_1 = 0.4\text{pg}^2/\text{mL}^2$ and $a_2 = 0.7\text{mL}^2/\text{n}^2$ seem to be prescribed rather than found through some iterative process. Furthermore, if such an iterative process was implemented, what is the convergence criteria for the iterative process?

Response: Choosing values of the weights a_1 and a_2 is a trade off between getting close to P_0 and not using too much cost. To determine optimal time-varying dosages, the authors initially assigned random values with order of magnitude between -2 and 1 as initial guesses for the weights. We constructed an iterative algorithm adjusting weights down if a given weight produced P_4 peak higher than 5 ng/mL. The iterative procedure was repeated until weights that make maximum $P_4(t) < 5$ ng/mL are obtained. To make this clearer we have revised the manuscript page 15.

Methods (Page 10, lines 309–312): Random values with order of magnitude between -2 and 1 are originally assigned as initial guesses for the weights. If these weights produced P_4 peak higher than 5 ng/mL, their values are adjusted down. This iterative procedure was repeated until weights that make maximum $P_4(t) < 5$ ng/mL are obtained.

(iii) To illustrate the effect of parameters a_1 and a_2 on the optimal solutions, it would be good to have for the single hormone, constant dosing cases plots of the optimal u_1 against a_1 and u_2 against a_2; this should help the reader understand better the role of a_1 and a_2 on the optimal solutions.

Response: Generally, in control theory increasing the weights makes the control values go down. We illustrate a few examples in Supplementary C. As suggested by the reviewer, we have added figures in Supplementary C depicting the effect of varying weights on the optimal solutions. As mentioned on page 24, paragraph 2, and page 26, paragraph 3, increasing weights penalizes the exogenous hormone dose causing larger deviation of maximum P_4 from the target point 4 ng/mL.

Supplementary C:

Supplementary C: Optimal exogenous hormones for different weights

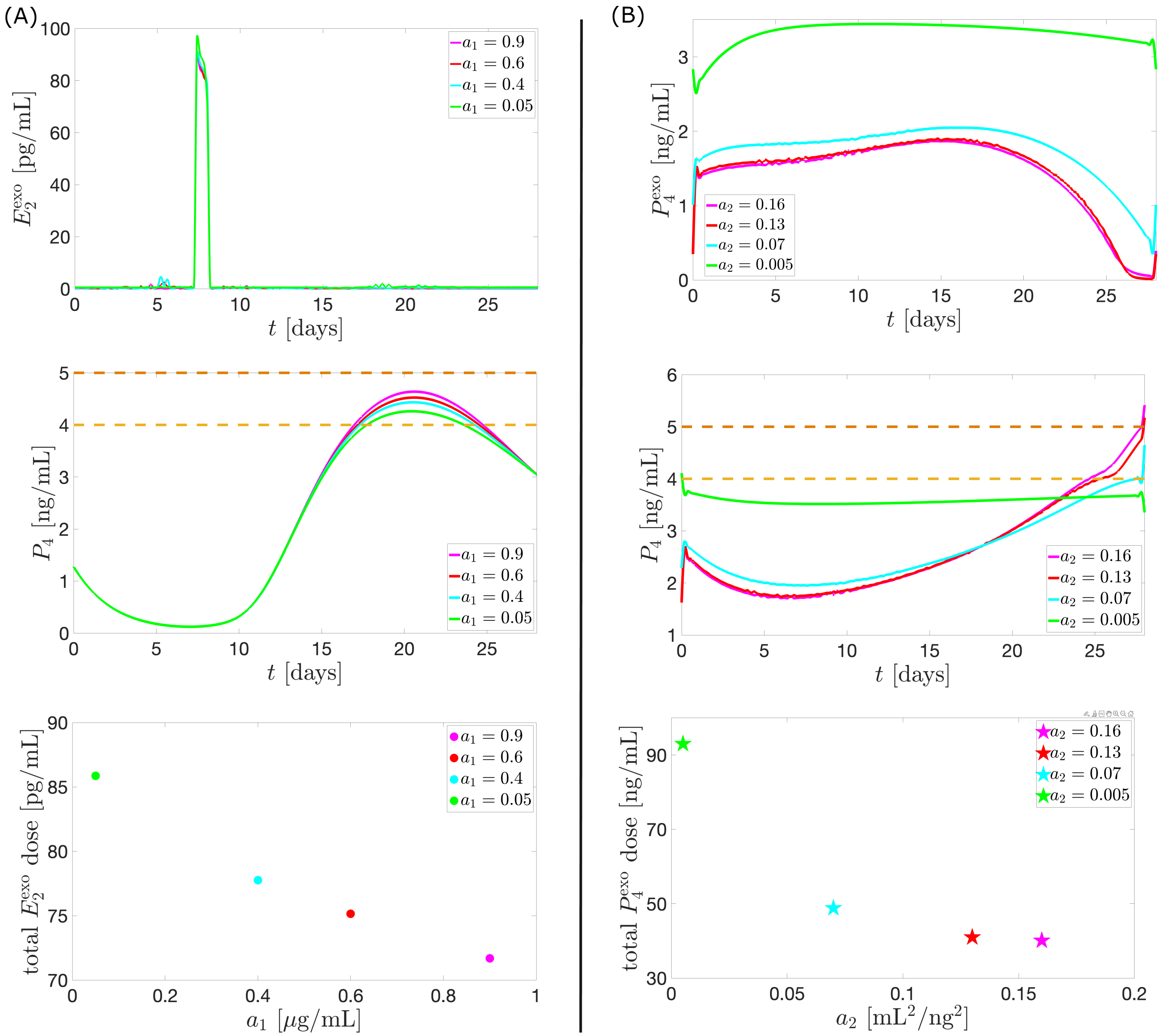

Figure : **Varying weight parameters in monotherapy.** Panel (A) shows the effect of varying the weight parameter a_1 in estrogen monotherapy on the optimal E_2^{exo}, and subsequently on the P_4 level and total exogenous estrogen dose. Panel (B) presents the effect of changing a_2 in progesterone monotherapy on optimal P_4^{exo}, on P_4 concentration, and total exogenous progesterone dose.

(iv) Putting together points (ii) and (iii), more explanation is needed on the choice of a1 and a2 in their optimisations.

Response: As suggested, another way to investigate the effect of weights a_1 and/or a_2 is to breakdown the objective function $\int_0^{28}\left((P_4(t)-4)^2+a_1u_1+a_2u_2^4\right)dt$ to its components $\int_0^{28}\left((P_4(t)-4)^2\right)dt$, $\int_0^{28}(a_1u_1)\,dt$, and $\int_0^{28}(a_2u_2)\,dt$ (see Supplementary E). This shows which component contributes more to the cost and in turn, provide guidance on how the weights can be adjusted to attain desired P_4 level.

(v) The authors' state "Very oscillatory results are obtained for power of u2 less than four". Is this saying that the "optimum" u_2 is oscillatory or is this in terms of convergence behaviour? Their solution to the problem seems a little arbitrary and it appears odd to me that the form of the cost terms for u1 and u2 are different. The discussion on this needs expanding.

Response: We have made an exhaustive investigation of the coefficients a_1 and a_2, and also the powers of u_1 and u_2 in the objective function, and the one which presented results with least oscillation is $\int_0^{28}\left((P_4(t)-4)^2+a_1u_1+a_2u_2^4\right)dt$. To identify the best objective function, we started with linear u_1 and u_2 cost terms. However, this form produced oscillatory results. Several runs revealed that the oscillations where the highest for optimal progesterone results. Thus, we increased the exponent of u_2 in the cost function and settled for a fourth power to smoothen the optimization results. Several outcomes from different forms of objective function are included in Supplementary D.

Methods (Page 10, lines 300–306): We have made an exhaustive investigation of the powers of u_1 and u_2 in the objective function which presented results with least oscillation (few examples are shown in Supplementary D). To identify the best objective function, we started with linear u_1 and u_2 cost terms. However, this form produced oscillatory results. Several runs revealed that the oscillations where the highest for optimal progesterone results. Thus, we increased the exponent of u_2 in the cost function and settled for a fourth power to smoothen the optimization results.

Supplementary D:

Supplementary D: Model output for variants of the objective function

1. Optimal exogenous estrogen and progesterone if the objective function is $\int_0^{28}\left((P_4(t)-4)^2+a_1u_1+a_2u_2\right)dt$.

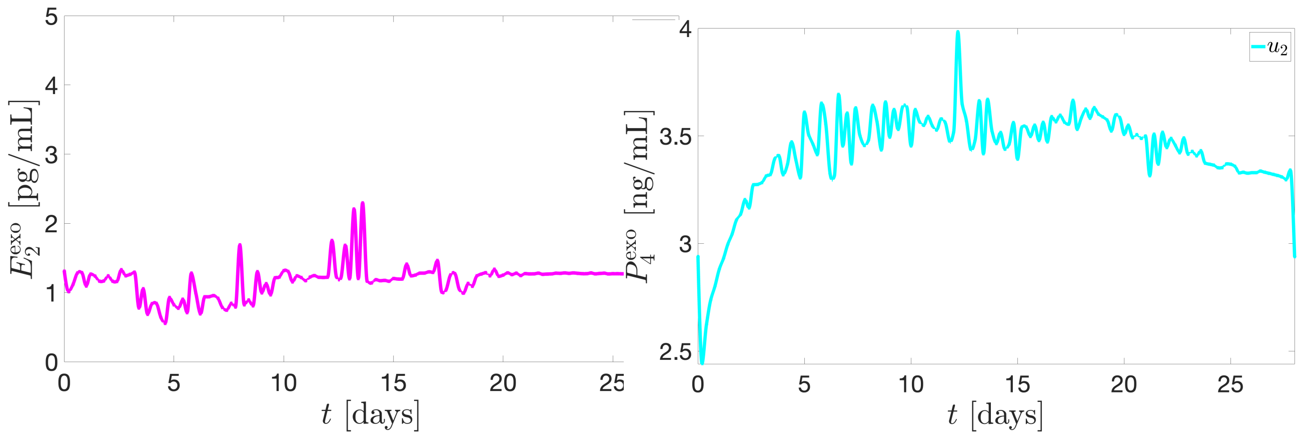

Figure : **Linear third term in objective function with $a_1 = 0.4$ and $a_2 = 0.7$.** Optimal exogenous estrogen (on the left) and optimal exogenous progesterone (on the right) if the objective function is $\int_0^{28}\left((P_4(t)-4)^2+0.4u_1+0.7u_2\right)dt$.

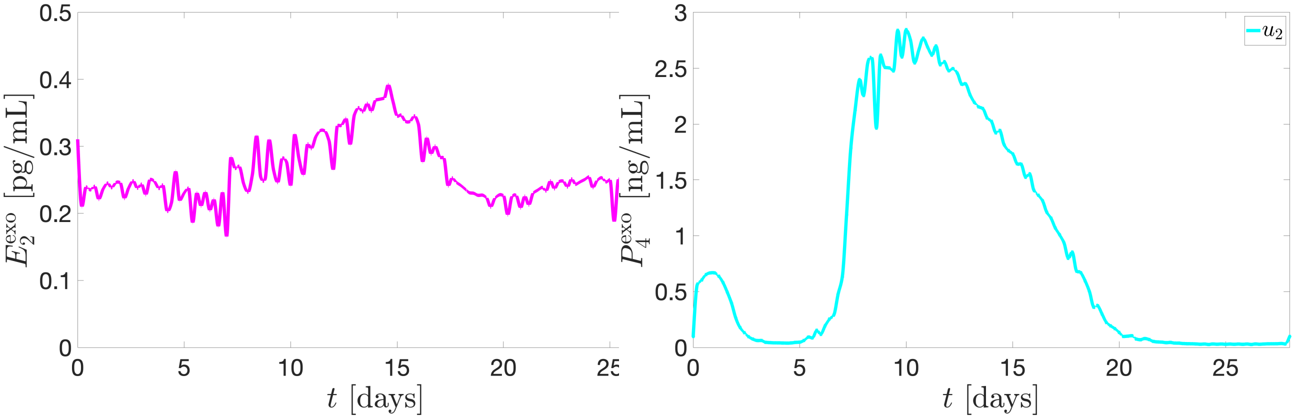

Figure : **Linear third term in objective function with $a_1 = 0.4$ and $a_2 = 2$.** Optimal exogenous estrogen (on the left) and optimal exogenous progesterone (on the right) if the objective function is $\int_0^{28} \left((P_4(t) - 4)^2 + 0.4u_1 + 2u_2\right) dt$.

2. Optimal exogenous estrogen and progesterone if the objective function is $\int_0^{28} \left((P_4(t) - 4)^2 + a_1u_1 + a_2u_2^2\right) dt$.

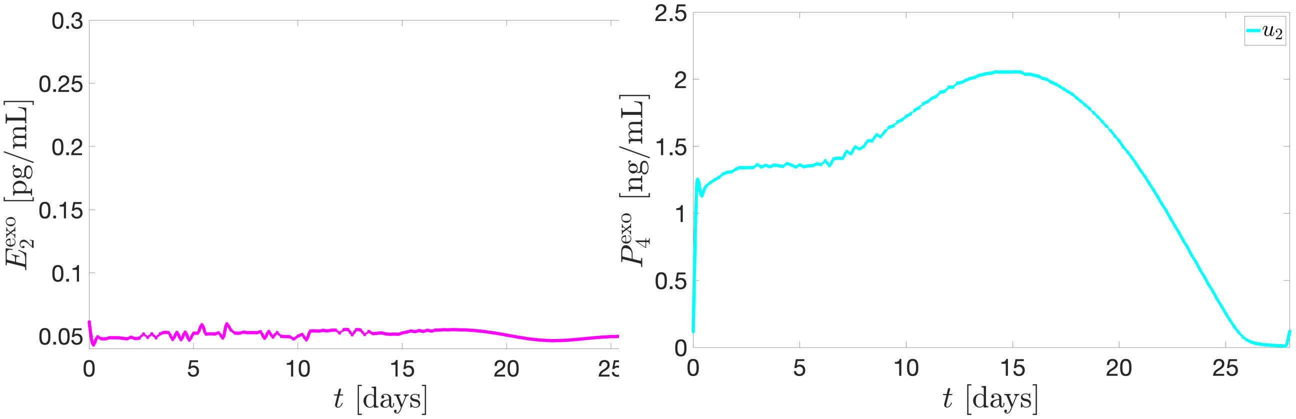

Figure : **Quadratic third term in objective function with $a_1 = 0.4$ and $a_2 = 0.7$.** Optimal exogenous estrogen (on the left) and optimal exogenous progesterone (on the right) if the objective function is $\int_0^{28} \left((P_4(t) - 4)^2 + 0.4u_1 + 0.7u_2^2\right) dt$.

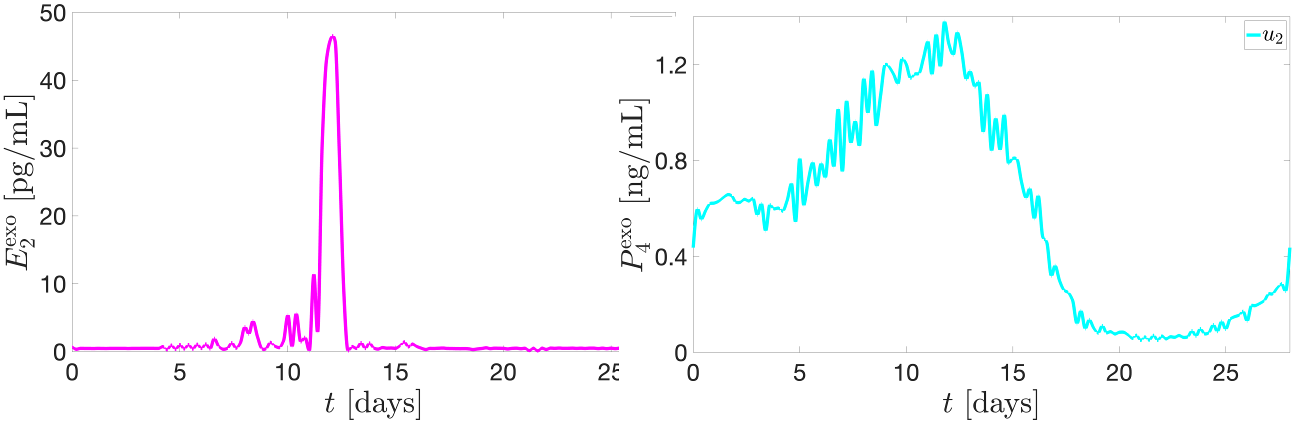

Figure : **Quadratic third term in objective function with $a_1 = 0.2$ and $a_2 = 2.5$.** Optimal exogenous estrogen (on the left) and optimal exogenous progesterone (on the right) if the objective function is $\int_0^{28} \left((P_4(t) - 4)^2 + 0.2u_1 + 2.5u_2^2\right) dt$.

3. Optimal exogenous estrogen and progesterone if the objective function is $\int_0^{28} \left((P_4(t) - 4)^2 + a_1 u_1 + a_2 u_2^3\right) dt$.

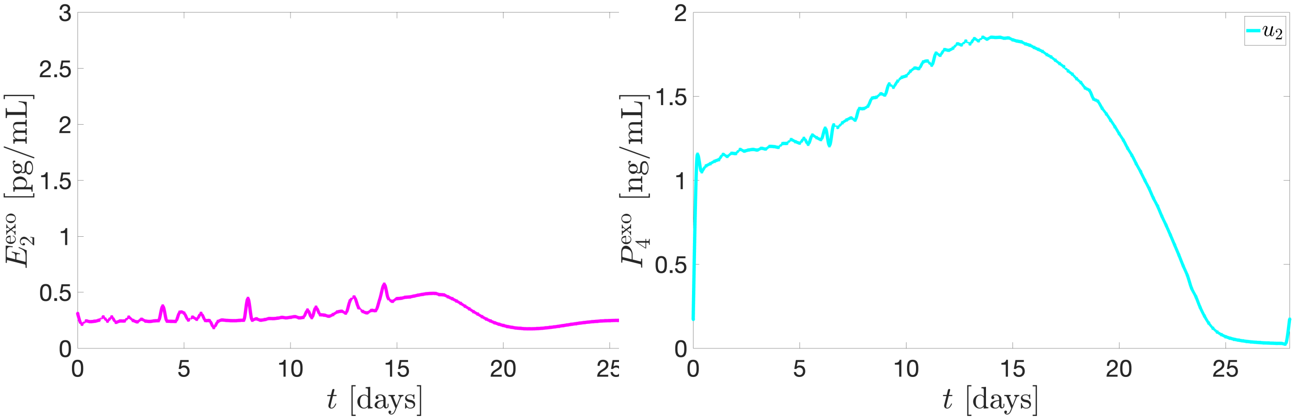

Figure : **Cubic third term in objective function with $a_1 = 0.4$ and $a_2 = 0.7$.** Optimal exogenous estrogen (on the left) and optimal exogenous progesterone (on the right) if the objective function is $\int_0^{28} \left((P_4(t) - 4)^2 + 0.4u_1 + 0.7u_2^3\right) dt$.

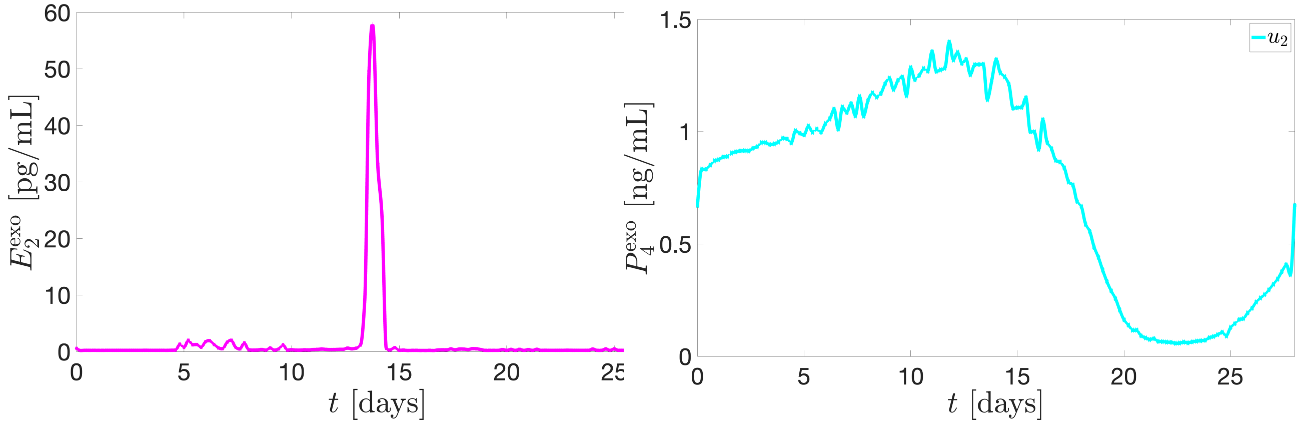

Figure : **Cubic third term in objective function with** $a_1 = 0.4$ **and** $a_2 = 1.5$. Optimal exogenous estrogen (on the left) and optimal exogenous progesterone (on the right) if the objective function is $\int_0^{28} \left((P_4(t) - 4)^2 + 0.4u_1 + 1.5u_2^3\right) dt$.

(vi) There seems to be a units mismatch in the function. The units of $(P_4(t) - P_0)^2$ is presumably ng^2/mL^2, but $a_1 u_1$ seems to be pg^2/mL^2. This needs fixing or explaining.

Response: The unit for $a_1 u_1$ is $(\mu\text{g/mL})(\text{pg/mL}) = \text{ng}^2/\text{mL}^2$ which matches the unit for the remaining two terms in the cost function.

(vii) In their optimum solutions, I do not have a sense whether the greatest cost in an optimised solution comes from the $\int_{t_0}^{t_f} (P_4(t) - P_0)^2 \, dt$ term, the other terms or that they are all about the same size. A brief "We found that ..." type statement on page 15 would be good to address this.

Response: Supplementary E presents a breakdown of the optimal cost for a few optimized solutions. Most of the weights produced bigger $\int_{t_0}^{t_f} (P_4(t) - P_0)^2 \, dt$ than the remaining two terms but the small weight $a_2 = 0.005$ resulted to a low value of $\int_{t_0}^{t_f} (P_4(t) - P_0)^2 \, dt$. This weight though results to a large total exogenous progesterone dose of 92 ng/mL (see Supplementary C). This further illustrates that choosing weights a_1 and a_2 is a trade off between getting near P_0 and not using too much cost.

Methods (Pages 10-11, lines 312–314):
The constants $a_1 > 0$ an $a_2 > 0$ are weight factors which balance the effort of minimizing u_1 and u_2, and hitting P_0. We found that low values of weights produce small value of $\int_{t_0}^{t_f} (P_4(t) - P_0)^2 \, dt$ (see Supplementary E), i.e., $P_4(t)$ is near P_0, but increases exogenous hormone dose.

Minor comments

1. Least squares formulation on Page 14. Why multiply the relative errors by a 100? This effectively multiplies the weighting terms by 10 and surely has no impact on the results.

   Response: The multiplication of relative errors by 100 indeed has no impact on the results and was done only as a preferred form. We have removed this multiplier in the cost function (page 14).

   Methods (Page 9, lines 257–259):

$$\frac{1}{M-N} \sum_{i=1}^{M} \left(w_{E_2^*}(i) \left((E_2(i) - E_2^*(i)) \frac{1}{E_2^*(i)} \right)^2 + w_{P_4^*}(i) \left((P_4(i) - P_4^*(i)) \frac{1}{P_4^*(i)} \right)^2 + \right.$$

$$w_{Inh^*}(i) \left((Inh(i) - Inh^*(i)) \frac{1}{Inh^*(i)} \right)^2 + w_{LH^*}(i) \left((LH(i) - LH^*(i)) \frac{1}{LH^*(i)} \right)^2 +$$

$$w_{FSH^*}(i)\left((FSH(i) - FSH^*(i))\frac{1}{FSH^*(i)}\right)^2\Bigg),$$

2. Figure 14 is very interesting. Close inspection of P_4 in Fig 14B suggests the optimal dosing solutions have period-doubled, with maximal P_4 peaks occurring on alternate cycles. Furthermore, it appears from fig. 14B that the optimal oestrogen doses are becoming equally spaced apart - is this correct? This should be mentioned in the discussion, since such dosing strategy would be much easier to prescribe for the user.

   Response: In the original manuscript, we investigated the optimal dosing solution from day 0 to day 112. We increased the time interval to 280 days to explore the case more and found that application of high-dosage portion of optimal exogenous estrogen u_1 in equal intervals from day 35 can't sustain anovulation, but if administration is triggered by a specific biomarker, here the E2 level, ovulation can continually be inhibited as depicted in Figure XXX. So if a smart device is developed, even if some patients' cycle length vary a little bit from month to month, their E_2 level can be measured to know when to make a contraceptive device spike.

   Discussion (Pages 17–18, lines 539–550):
   For instance, if the large-dose portion of u_1 is applied in equal intervals from day 35 to day 280 (see Figure 15, anovulation is no longer induced beginning on the 5th 28-day period (i.e. from day 140 to day 280). This is because the application of u_1 in the preceding period changes the dynamics of the menstrual cycle in the succeeding days. One of these changes is the cycle length. Now, because the control u_1 must be administered at a time before E_2 surge (when u_1 is not applied), the timing of administration in the next cycles must also be changed to continuously suppress ovulation. Sometimes resulting cycle length is less than 28 days so only a portion of u_1 will be applied. In addition, because lower levels of u_1 are negligible compared to the higher levels, we applied only the large-dose portion of u_1 from day 35 to day 280 (see Figure XXX). The administration is done when E_2 level is increasing and reaches 75 pg/mL. We are currently conducting further investigation to determine the highest E_2 hormone level where administration of u_1 would commence to still inhibit ovulation. Note that although u_1 is applied eighteen times (in unequal intervals) from day 0 to day 280, the total dosage is still significantly lower than the total constant dosage of exogenous E_2 which induces anovulation. The dosing regimen presented, where administration is triggered by a specific biomarker, here the 75 pg/mL-E_2 level, offers insights on construction of timed devices that give contraception at certain parts of the menstrual cycle. For instance, contraceptive dose may be given relative to E_2 level. So even if some patients' cycle length vary a little bit from month to month, their E_2 level can be measured to know when to make a contraceptive device spike.

   Discussion (Page 18, Figure 14):

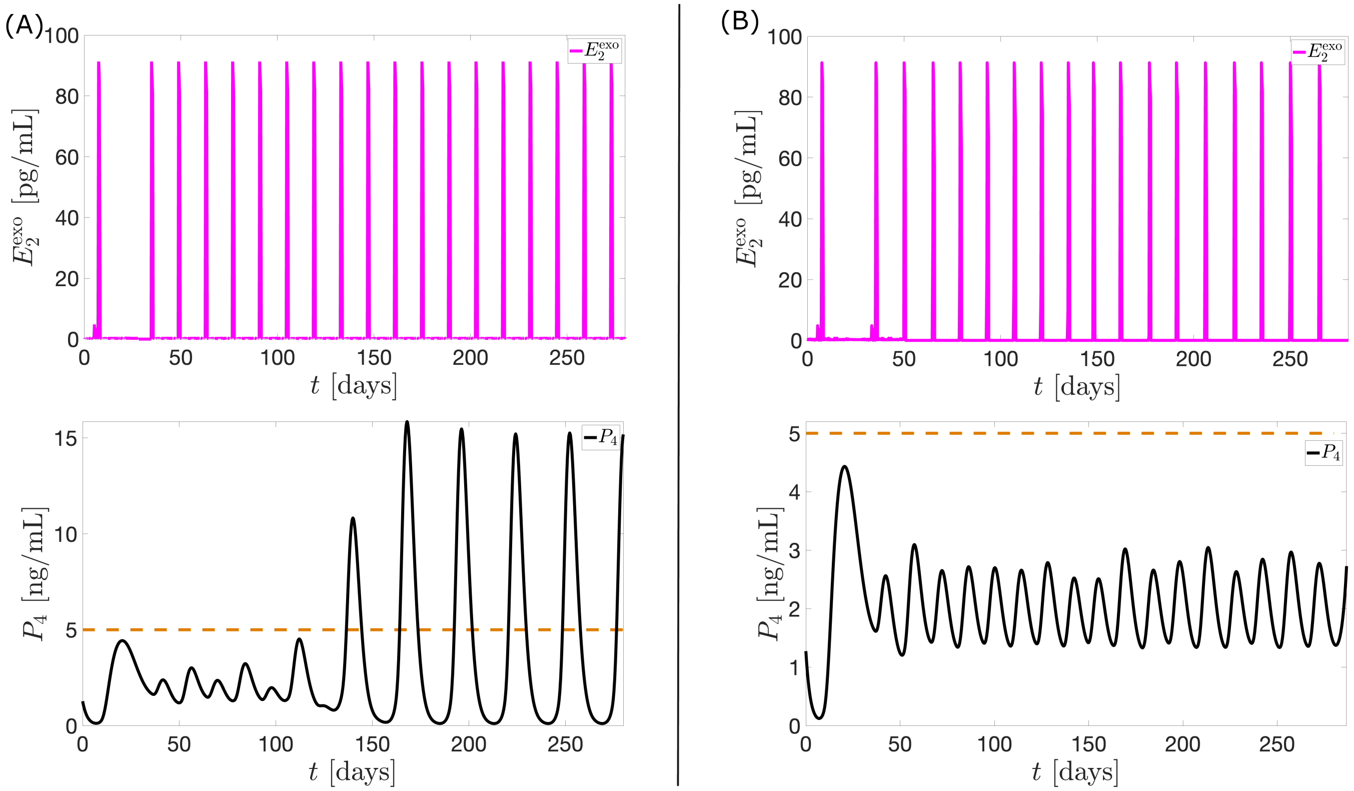

Figure : Multiple application of optimal control u_1. The black curve is the model output when multiple u_1 (magenta curve) is applied. In panel (A), the application of u_1 from day 35 to day 280 in equal intervals is unable to sustain anovulation. Panel (B) shows a scheme for administration of multiple u_1 which continuously blocks ovulation.

REFEREE 3

We thank the reviewer for carefully reading our manuscript providing constructive suggestions for edits. We have addressed all your comments below indicating changes that we have made to the manuscript in blue.

Minor comments:

1. I think the literature survey for applications of optimal control in biology is limited. For example, a recent book entitled "Control Applications for Biomedical Engineering Systems" academic press(2020) provides several examples, including optimal control related to hormonal treatments. I urge the authors to include all this recent literature.

   Response: We thank the reviewer for pointing out this deficiency, we have enriched the literature including additional references. We added the following phrases in the Introduction:

   Introduction (Page 3, lines 81–84):
   Several studies explore optimization of hormonal treatments. For instance, [10] described an optimal dosing regimen for the infusion of FSH to patients undergoing in vitro fertilization. While in [11], control theory is employed to investigate optimal dosage decisions in the administration of gonadotropin in controlled ovarian hyperstimulation treatment cycle.

2. The authors describe the model in Figure 3 but do not provide any information about control variables $u_1(t) = E_2^{\text{exo}}(t)$ and $u_2(t) = P_4^{\text{exo}}(t)$ in the figure. Please include that so that one can understand the control problem.

   Response: The control variables $u_1(t) = E_2^{\text{exo}}(t)$ and $u_2(t) = P_4^{\text{exo}}(t)$ have been included in the model diagram in Figure 3. The revised figure is shown below.

   Methods (Figure 4):

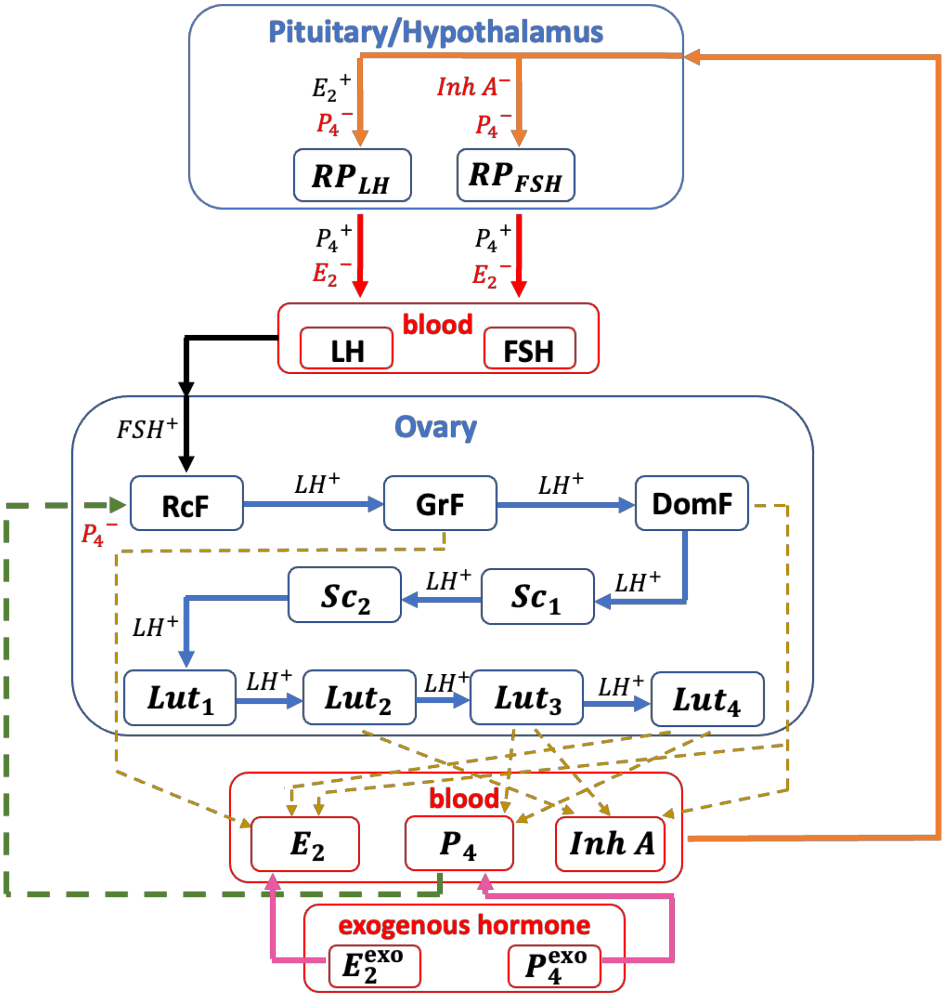

Figure : **Model diagram.** The diagram (adapted from [12]) shows the 13 states (RP_{LH}, RP_{FSH}, LH, FSH, RcF, GrF, $DomF$, Sc_1, Sc_2, Lut_1, Lut_2, Lut_3, Lut_4) in the menstrual cycle model. H^+ denotes stimulation by hormone H while H^- denotes inhibition. The red arrow presents the release of the pituitary hormone from the reserve pool to the blood. The black arrow indicates the influence of the pituitary system on the ovarian system while the orange arrow denotes the ovary's feedback. The green dashed arrow denotes inhibition of P_4 in the RcF stage. The blue arrow desribes the transition of a follicle from one ovarian stage to the next. The gold dashed arrow denotes the state contributing to the ovarian hormone production. The pink arrow represents the infusion of exogenous hormone to the ovarian system.

3. In the results section, the authors present the residuals for each variable in Table II separately. There is an order of magnitude difference between variables. How did they combine the objective function of residuals (to be minimized) in their analysis? Please provide details.

   Response: On Page 14, the function used to minimize the least squares error is a combination of the percentage error for each hormone. This eliminates the order of magnitude difference between the variables. We have added the following text.

   Methods (Page 9–10, lines 271–274)

   Because the hormones have different units, the multiplier $\frac{1}{H^*(i)}$ is used to obtain the term $(H(i) - H^*(i))\frac{1}{H^*(i)}$, the percentage error for hormone H. This eliminates the order of magnitude difference between the variables, allowing addition of residuals in the objective function.

4. They do not explain how they obtained results for optimal constant dosage. What type of optimization did they use?

   Response: We obtained results for optimal constant dosage as follows:

   Results (Page 12, lines 379–380):
   To determine $E_2^{\text{exo}}(t)$ and $P_4^{\text{exo}}(t)$ values which block ovulation, we observe the LH and P_4 model output as the dosages vary from 0 to 60 pg/mL per day on a 0.1 pg/mL-interval for $E_2^{\text{exo}}(t)$, and 0 to 4 ng/mL per day on a 0.1 ng/mL-interval for $P_4^{\text{exo}}(t)$.

   Results (Page 14, lines 410–416):
   To determine optimal constant dosage resulting in anovulation, P_4 output peaks are recorded for each combination of $E_2^{\text{exo}}(t)$ and $P_4^{\text{exo}}(t)$ dose. For the estrogen monotherapy, the dosage of E_2^{exo} combined with $P_4^{\text{exo}} = 0$ (dosage of P_4^{exo} combined with $E_2^{\text{exo}} = 0$ for progesterone monotherapy) resulting in P_4 peak of 4.99 ng/mL is taken as the optimum constant dosage. In the combined therapy, the optimum constant dosage is the nonzero dosage of E_2^{exo} combined with nonzero P_4^{exo} resulting in P_4 peak of 4.99 ng/mL.

5. They talk about computational intensity but do not provide any details. Please provide the details of computational time.

   Response: We always get convergence in the simulations and computation time varies from a few hours to several days, depending on the proximity of initial guess to the optimal solution. We have added a table listing examples of computational time for particular cases in Supplementary E. In the future, one may want to study the computation time using parallel processing, running several initial guesses at the same time, but at present investigating the algorithm is not the emphasis of this work.

   Discussion (Page 16, lines 497–498):
   Computation time ranges from few hours to several days depending on the proximity of initial guess to the optimal solution. Supplementary E illustrates some examples.

   Supplementary E:

   Supplementary E: Computation time and optimal cost

Table : **Computation time and optimal cost.** Computation time (T) and optimal cost (J) for the three therapies. Estrogen monotherapy (Est Mon), Progesterone monotherapy (Prog Mon), and the combination therapy (Comb). All therapies use a time-step $\Delta t = 0.2$. A breakdown of the optimal cost into the three integrals (Int 1, Int 2, and Int 3) comprising the objective function is also presented.

**Treatment**	**Objective function:** $\int_0^{28} \left((P_4(t)-4)^2 + a_1u_1 + a_2u_2^4\right) dt$	**T (s)**	**J**	**Int 1**	**Int 2**	**Int 3**
Est Mon	$a_1 = 0.9$; $a_2 = 0$	249067	236.07	171.55	64.52	0
Est Mon	$a_1 = 0.6$; $a_2 = 0$	192656	216.28	171.19	45.09	0
Est Mon	$a_1 = 0.4$; $a_2 = 0$	103044	202.02	170.92	31.10	0
Est Mon	$a_1 = 0.05$; $a_2 = 0$	274871	174.98	170.69	4.29	0
Prog Mon	$a_1 = 0$; $a_2 = 0.16$	31840	105.31	76.39	0	28.92
Prog Mon	$a_1 = 0$; $a_2 = 0.13$	198838	99.67	74.33	0	25.34
Prog Mon	$a_1 = 0$; $a_2 = 0.07$	13498	85.83	64.03	0	21.80
Prog Mon	$a_1 = 0$; $a_2 = 0.005$	59277	22.17	4.92	0	17.25
Comb	$a_1 = 0.4$; $a_2 = 0.7$	344799	137.19	106.59	14.23	16.38

Int 1 $= \int_0^{28} \left((P_4(t)-4)^2\right) dt$
Int 2 $= \int_0^{28} \left(a_1u_1\right) dt$
Int 3 $= \int_0^{28} \left(a_2u_2^4\right) dt$

Additional revision from authors

Table : **Model output peaks and sum of squared residuals between the model output and Welt data.**

Hormone	Sum of squared residuals	Hormone peak		
		Hormone	Welt data	Model output
E_2	21839.63 (in pg^2/mL^2)	E_2 (in pg/mL)	237	237.74
P_4	182.77 (in ng^2/mL^2)	P_4 (in ng/mL)	17.9	18.06
Inh	66.55 (in IU^2/mL^2)	Inh (in IU/mL)	11.5	11.47
LH	1579.46 (in IU^2/L^2)	LH (in IU/L)	123	123.12
FSH	174.19 (in IU^2/L^2)	FSH (in IU/L)	19.6	20.36

We have added title to the figure captions in the Results and Discussion sections.

Results (Figure 4):
Fig 4. **Normal cycle solution.**

Results (Figure 5):
Fig 5. **Model output with constant dose of exogenous hormone.**

Results (Figure 6):
Fig 6. **Varying E_2^{exo} dose.**

Results (Figure 7):
Fig 7. **Varying P_4^{exo} dose.**

Results (Figure 8):
Fig 8. **Constant dose combination therapy.**

Results (Figure 9):
Fig 9. **Model output with application of optimal control u_1.**

Results (Figure 10):
Fig 10. **Follicular mass with application of optimal control u_1.**

Results (Figure 11):
Fig 11. **Model output with application of optimal control u_2.**

Discussion (Figure 12):
Fig 12. **Model output with application of optimal controls u_1 and u_2.**

Discussion (Figure 13):
Fig 13. **Constant dosage and nonconstant dosage comparison.**

Discussion (Figure 15):
Fig 15. **Multiple application of optimal control u_1.**

We changed some parameter units in the Supplementary material.

Supplementary A:
parameter: b unit: $L\mu g(IUday)^{-1}$
parameter: w unit: ng/mL
parameter: q unit: ng/mL

References

[1] Welt CK, McNicholl D, Taylor A, Hall J. Female reproductive aging is marked by decreased secretion of dimeric inhibin. J Clin Endocrinol Metab. 1999;84(1):105-11.

[2] Bormann I. DigitizeIt; 2021. ver. 2.5. Available from: https://www.digitizeit.xyz/.

[3] fminsearch. Matlab. Mathworks Inc. 2022.

[4] Margolskee A, Selgrade J. Dynamics and bifurcation of a model for hormonal control of the menstrual cycle with inhibin delay. Math Biosci. 2011;234(2):95-107.

[5] Lewis FL, Vrabie D, Syrmos VL. Optimal control. 3rd ed. John Wiley & Sons; 2012.

[6] Ferreira-Poblete A. The probability of conception on different days of the cycle with respect to ovulation: an overview. Advances in Contraception, volume=13, pages=83–95, year=1997.

[7] Royston P. Identifying the fertile phase of the human menstrual cycle. Statistics in Medicine. 1991;10(2):221-40.

[8] Lynch CD, Jackson LW, Buck Louis GM. Estimation of the day-specific probabilities of conception: current state of the knowledge and the relevance for epidemiological research. Paediatric and Perinatal Epidemiology. 2006;20(s1):3-12.

[9] Stirnemann JJ, Samson A, Bernard JP, Thalabard JC. Day-specific probabilities of conception in fertile cycles resulting in spontaneous pregnancies. Human Reproduction. 2013;28(4):1110-6.

[10] Azar AT. Control Applications for Biomedical Engineering Systems. Academic Press; 2020.

[11] He M, Zhao L, Powell WB. Optimal control of dosage decisions in controlled ovarian hyperstimulation. Annals of Operations Research. 2010;178(1):223-45.

[12] Wright A, FN G, Selgrade J, Olufsen M. Mechanistic model of hormonal contraception. PLOS Comp Biol. 2020 06;16(6):1-23.
