# Supplementary material for: Toward an optimal contraception dosing strategy
Source: PLoS Comput Biol. 2023 Apr 13;19(4):e1010073. doi: 10.1371/journal.pcbi.1010073 (PMC10101497; doi:10.1371/journal.pcbi.1010073)
Supplement: Attachment — Submitted filename: Response to Reviewers.pdf [file pcbi.1010073.s005.pdf]

# Toward an optimal contraception dosing strategy

Brenda Lyn A. Gavina, Aurelio A. de los Reyes V, Mette S. Olufsen, Suzanne Lenhart, Johnny T. Ottesen

RESPONSE TO REVIEWER'S COMMENTS

February 22, 2023

## REFEREE 1

We thank the reviewer for carefully reading our manuscript providing constructive suggestions for edits. We have addressed all comments below indicating changes to the manuscript. Our response is written in red and changes incorporated in the manuscript is listed in blue.

### Major comments

- The objectives of minimizing the exposure to both exogenous estrogen and exogenous progestin over time in order to keep progesterone below a fixed threshold are clearly stated. Some additional useful explanations are now added regarding the influence of the second and third terms in the integrand of the objective function J(u), as well as the choice of the $P_0$ value. The main assumption here is that the two exogenous compounds are strictly identical to the endogenous hormones and that the former are simply modifying the levels of the latter. It represents a very strong assumption which remains very far from the current reality.

  **Response:** As stated in the Introduction, the objective of this study is to add new developments to the state-of-the-art fundamental mathematical models for the female menstrual cycle and the transition to a contraceptive state. To make the specific assumptions for the underlying model clearer, we have added the following text to the Introduction:

  **Introduction (Pages 3-4, lines 89-102):** The current paper expands previously published papers by Selgrade et al. on the hormonal regulation of the menstrual cycle [1, 2] and the transition to contraception [3]. In this study, the model in Margolskee and Selgrade [4] is modified to include mechanisms depicting the contraceptive effect of exogenous progesterone on the menstrual cycle. This new model shows the principal mechanisms behind transition to contraception. It is calibrated to the patient-data extracted from Welt et al. [5] and predicts the daily levels of pituitary hormones $LH$ and $FSH$, and ovarian hormones $E_2$, $P_4$, and $Inh$ averaged during a normal menstrual cycle of 23 women. The model output also predicts reduction in pituitary and ovarian hormone levels caused by exogenous estrogen and/or progesterone observed by Obruca et al. [6] and Deb et al. [7].

  This paper uses an optimal control approach to simulate contraception using the model described above. The objective is to identify strategies to understand when and how much estrogen and/or progesterone to administer to obtain a contraceptive state.

  **Response:** We agree with the Reviewer that it is likely that exogenous hormones function differently from endogenous hormones. Our objective is to continue the series of studies by Selgrade et al. who used Equations (14) and (15). We recognize that these equations assume that exogenous doses of estrogen and progesterone act as endogenous hormones. To account for the fact that there may not be a one-to-one relation between exogenous and endogenous hormones, we add constants $b_1$ and $b_2$.

We do not have data for these parameters. We chose to let $b_1 = b_2 = 1$, devising a model that agrees with the original Selgrade et al. model [1, 2, 3] which was previously published and shown to work. To emphasize the point by the reviewer, in the Materials and methods section we modified Equations (14) and (15) and added explanation:

**Materials and methods (Pages 7-8, line 221):**

$$E_2(t) = e_0 + e_1 GrF(t) + e_2 DomF(t) + e_3 Lut_4(t) + b_1 E_2^{\text{exo}}(t) \quad (14)$$

$$P_4(t) = p_0 + p_1 Lut_3(t) + p_2 Lut_4(t) + b_2 P_4^{\text{exo}}(t) \quad (15)$$

**Materials and methods (Pages 7-8, line 221):** The constants $b_1$ and $b_2$ in Equations 14 and 15 reflect the functions of exogenous estrogen $E_2^{\text{exo}}(t)$ and progesterone $P_4^{\text{exo}}(t)$, given as blood concentrations, which may be different from endogenous hormones. The exact influence of the exogenous hormones on the endogenous hormone levels are not incorporated and thus we let $b_1 = 1$ and $b_2 = 1$ agreeing with the mathematical model published in [1, 2, 3].

- **Results:** The reviewer acknowledges the explanation given by the authors about the origin of the dataset, which are now directly available. However it remains a purely artificial ideal averaged endocrine profile, scaled a posteriori according to the day of ovulation and not to the individual starting times of the menstrual cycles of the women who contributed to the study by Welt et al. Repeating four times an unique averaged profile does not give any information about the within-between women's variabilities. It adds a spurious 28 days periodicity which is clearly not natural.

  **Response:** To our knowledge, publicly available human/female data are limited. We have not found any experimental studies reporting the dynamics during the transition to contraception, the topic of this study. The data included here are used to demonstrate that the core model behaves appropriately. We used the same dataset (to our knowledge one of the only complete cycle datasets from humans) to calibrate the model. These data present average over 23 normally cycling female centered to ovulation satisfying three of the following four criteria: i) LH peak day, ii) midcycle FSH peak day, iii) the day of or after the midcycle $E_2$ peak, and iv) the day marking doubling of $P_4$ from baseline or that the $P_4$ exceeds 0.6 ng/mL [5]. This was done to compare the dynamics across the follicular and luteal phases. The objective of our study is to demonstrate a principle, that by adding the proposed mechanisms it is possible to generate a contraceptive state. It is clearly possible to use animal data as suggested by the reviewer, but these would require careful attention to the mechanisms as they likely differ from humans. We find that this would shift the focus of the manuscript and therefore we have chosen to not pursue this approach. To make it clearer we have added the following text noting that the data are aligned to the day of ovulation. The revised text states:

  **Methods (Page 6, lines 170-177):** The Welt study [5] reports that the data are centered to the day of ovulation requiring three of the following four criteria to be satisfied: i) LH peak day, ii) midcycle FSH peak day, iii) the day of or after the midcycle $E_2$ peak, and iv) day that the $P_4$ doubled from its baseline or reached a level of 0.6 ng/mL. Before averaging the data the menstrual cycle data were standardized to a 28-day cycle length with the day of ovulation centered to day 0 and the mean hormone levels were averaged over the early, mid-, and late follicular phase and early, mid- , and late luteal phase, see [5] for more details.

  **Materials and methods (Page 9, lines 274-276):** The Welt data was standardized to 28 days. We used four repetitions of this data as shown in Figure 4 to obtain $M$ data points utilized in parameter estimation. This is done to obtain a periodic solution.

  **Discussion (Page 16, lines 487-491):** Biologically, the period length vary within people and between people. This work does not account for this variation, though it can be introduced by

varying model parameters or embedding the model in a stochastic framework. This limitation was introduced to generate a simple model that show that contraception can be obtained by manipulating certain parts of the menstrual cycle.

**Results (Page 12, lines 369-370):** It presents four cycles showing periodicity of the model output.

- Case 1 appears unrealistic, yet an interesting, theoretical exercise of long term administration of estradiol. Unfortunately, such an administration will most probably induce a growth of the endometrium exposing to hyperplasia and bleeding. It justifies the need from time to time to leave an open window without estrogen to allow endometrial bleeding, though the choice of the frequency of these off- windows remains completely free.

  It is interesting to note that exposing female rhesus monkeys to a constant intra-venous infusion of a low dose of estradiol has been experimented. It resulted in a complete shut down of the GnRH pulse generator which lasted several days or weeks (see for instance Ordog and Knobil, PNAS, 1995, 92, 5813-5136; O'Byrne and Knobil, Hum Reprod, 1993, 8(S2), 37-40). However endometrial bleeding remains very limited in this species.

  Restraining the administration of estradiol to shorter periods and varying regimens might expose to different effects not totally predictable depending on the degree of maturity of the existing developing follicles.

  **Response:** We appreciate these comments by the reviewer, to demonstrate the limitations of the model presented here, we have added text to the discussion addressing these points. The revised text reads:

  **Discussion (Page 17, lines 530-538):** A constant intravenous infusion of a low dose of estradiol result in complete shut down of the GnRH pulse generator on female rhesus monkeys for several days or weeks [8, 9]. Although endometrial bleeding is rare in this species, a constant administration may induce a growth of female human endometrium exposing to hyperplasia and bleeding. It justifies the need for an open window without estrogen to allow endometrial bleeding. The optimization result for the time-varying administration of estrogen suggests a dosing regimen generating an estrogen-free window. As is the case with new drug regimen, clinical studies would further assess the effects of restraining the administration of exogenous estrogen to shorter periods and varying regimens.

- Case 2. It assumes that natural progesterone can be given exogenously with a constant concentration level over the 24- hour period. When started at the end of the previous luteal phase it should theoretically induce an inhibition of FSH release by the gonadotropin cells, and hence hinder the follicular growth with all the consequences on estradiol secretion and absence of ovulation. It is clear that this regimen has to be maintained continuously as any sharp drop in the progesterone level will be followed by an increase in FSH and a resumption of the late stage of follicular growth.

  Progestins are clearly different in their effects on the hypothalamo-pituitary ovarian axis as compared to natural progesterone and their effects are not uniform depending on their androgenic effect and their respective metabolism. Hence a large part of the contraceptive effect of progestin- alone contraceptive, either given orally or as implant relies on their effects on the vaginal secretion and the tubal ciliary mobility inhibition. As the follicular growth and the ovulation is not constantly prevented, any disruption in the intake exposes to unwanted pregnancies with a higher risk of ectopic pregnancy.

  **Response:** We appreciate these comments by the reviewer, to demonstrate the limitations of the model presented here, we have added text to the discussion addressing these points. The revised text reads:

  **Discussion (Page 17, lines 545-549):** Exogenous progesterone like progestin may affect the hypothalamo-pituitary ovarian axis differently compared to endogenous progesterone. The current model shows a principle of contraception with exogenous administration of progesterone. If the

influence of a specific exogenous progesterone is represented, the current model should be coupled with a pharmacokinetics model that describes the details of the drug.

- Case 3. The use of a combined administration corresponds to the majority of pill proposed on the market, although not with natural compound except for some recent pill using estradiol valerate as the estrogenic component. The originality of the work is to adjust the administration profile of both estradiol and progesterone to limit the total progesterone concentration at any time. The analysis of the outcome of the model is rather complex to understand from the physiological standpoint although it complies with the behavior of the proposed deterministic model.

  After a few pill cycles, it remains hard to speak about a follicular phase and a luteal phase as they do not exist anymore. However a follicular growth persists up to a follicular stage where the FSH receptors are active. The suggestion of delaying the administration of estrogen and to limit it to the mid- pill cycle exposes to an abrupt decrease in the estrogen level which may stimulate a rise in FSH with the consequence of selecting a follicle ending up with an ovulation. An increase in the amount of the exogenous natural progesterone at later stage as indicated might have the opposite effect of favoring the implantation of a fertilized ovum.

  **Response:** In this work we show that a 28-day period, which formerly presents ovulation, no longer exhibits ovulation due to administration of combined exogenous estrogen and progesterone. The result suggests that estrogen administration should be coupled with an administration of progesterone at the start of the cycle. If only estrogen is given as shown in Case 1, estrogen should be given earlier, before the 10th day of the cycle. To clarify this point, we modified the text in the discussion.

  **Results (Page 16, lines 470-475):** Our simulation shows that the sole administration of estrogen (see Figure 9(A)) blocks ovulation if it is done prior to the 10th day of the cycle. Interestingly, the combination therapy (see Figure 12(A)) suggests that time-varying doses of estrogen and progesterone given simultaneously from the start to the end of the 28-day period, only requires a surge in estrogen dose around the 12th day of the cycle (a delayed administration compared to the estrogen monotherapy).

- **Significance of the biological findings:** The reviewer reemphasizes that the reproductive function in women is a very complex multiscale dynamical system highly exposed to both endogeous and exogenous factors. It explains both the large intra and between women variability of the menstrual cycle length over a reproductive period. The reviewer remains very concerned by a limited discussion about this aspect as the only dataset used by the authors concern daily mean values of measured blood concentrations of pooled menstrual cycles in normally cycling women.

  The authors proposes a sensitivity analysis which does not fully address the intrinsic random aspects of the endocrine cycle, as the only relative "constant" part of the cycle appears to be the duration of the luteal phase when an ovulation occurred, whereas the process of selection of the dominant follicles and its subsequent growth induces a more random follicular phase duration.

  The claim that this modelling represents a proof of concept that could be adapted to individual profiles is in theory correct but obtaining this daily blood profile even for one single cycle in routine appears totally unrealistic for both financial and practical reasons.

  Suggesting that the administration regimen could be triggered by estradiol blood concentration appears as a still futuristic and unrealistic perspective. It appears at least very remote of what is the current concern of access to contraception for all the women whatever their socioeconomic status is.

  The goal of any contraceptive method, whatever is the method, is to insure a protection against an unwanted pregnancy over a period of time, not restricted to a single menstrual cycle with women exposed to various environments, including stresses, infections, acute metabolic conditions, .... The present manuscript does not really address this very important issue.

**Response:** We acknowledge the comment by the reviewer emphasizing that optimizing any realistic model to repeated cycles with the present technology is futuristic in nature. As noted earlier, the objective of this study is to demonstrate a principle, that optimal control can identify the transition to contraception. The principles put forward in this study could easily be adapted to the analysis of data from individual subjects, and even though it would be computationally challenging the techniques can easily be expanded to the analysis of multiple cycles. To our knowledge, in humans multi-cycle data do not exist. Therefore, we find that adding this component is beyond the scope of this study. Moreover, while our model does not capture all factors in contraception, with emergence of more data, it can be refined to address other contraception issues. The suggestion by the reviewer is excellent, and therefore we have added a comment to this end encouraging future modelers and experimentalists to consider this point.

**Discussion (Page 19, lines 608-620):** The reproductive function in women is a very complex multiscale dynamical system highly dependent on both endogenous and exogenous hormones thus, the model developed in this study does not capture all factors involved with contraception. Rather, the model serves as a first step in using mathematical modeling to study the transition to a contraceptive state. When more data on individual hormone variations become available, the approach used here can be extended to use these. Another direction is to couple the model with a pharmacokinetics model to obtain patient-specific model to investigate the effects of specific contraceptives on individual menstrual cycle conditions. This provides avenue to examine further the complex multiscale effects of the factors included in silico. While at present, obtaining daily blood profile could be challenging both for financial and practical reasons, this study may motivate future development of advanced methodologies and technologies in data collection.

**Discussion (Page 18, lines 588-589):** This is analogous to the study where $LH$ levels are used as indicator for the time of antagonist administration in GnRH antagonist protocols [10].

- Furthermore, several strong assumptions are made which limit the conclusions of the authors. The explanations given in their rebuttal to the three assumptions, i.e. both within- and between- women's variabilities, modification in the dosage and timing along time, failure of taking into account the exact nature of the administered hormones and their metabolisms either alone or combined are not enough caution about their consequences on the interpretation of their results.

  Response: We agree with the reviewer that these are valuable points, which could be included in future mathematical models. We have added the following text to the conclusion:

  **Conclusion (Pages 19-20, lines 637-641):** In future studies, researchers should consider stochasticity in the model to investigate within- and between- women's variabilities and couple the current model with a pharmacokinetics model to take into account the exact nature and metabolisms of administered hormones, allowing investigation into effects of specific drugs.

- Hence any suggestion of shortening the duration of exposure to exogenous steroids exposes to the risk of unwanted pregnancies. This has been unfortunately largely reported with the consequence of always shortening the pill- off period and not extending it. The exact nature of the steroid compounds is also very important both in terms of actions on the steroid receptors and in terms of metabolism. Natural hormones cannot be so easily used so far for contraception. Any change of dosage along the cycle exposes to the risk of confusion in the daily intake, unless depending on a automatized rather sophisticated delivery system which again appears so far unrealistic.

  **Response:** These comments are clearly important and relevant. However, the objective of this work is to study the mechanism in principle continuing to expand the state-of-the-art models. As noted previously, these more specific considerations will be referred to future studies. We add in the Conclusion:

  **Conclusion (Page 19, lines 631-633):** With the advent of fully automated hormone-delivery

device like the intravaginal prototype device for cattles [11], continuous hormone administration to significantly reduce exogenous hormone dosages in humans is of interest.

### Minor comments

- page 17-23, the paragraph "Hormonal contraception benefits go beyond contraception. Estrogen also decrease motor skills, reducing the normal neurosmuscular protective mechanisms of the knee" does not appear very relevant to the present work and could be easily suppressed. Intensive training more or less associated with severe nutritional restriction expose indeed to hypo-estrogenemia and its pathological consequences. Indeed, an adapted hormonal contraception bringing back a normo-estrogenic state alleviates these effects, without the need of a sophisticated modelling approach.

  **Response:** Thank you for the suggestion, we have already removed from the Discussion section the text about benefits of hormonal contraception in ACL injury. We have added in the Introduction section:

  **Introduction (Page 3, lines 52-60):** Aside from contraceptive benefit, suppression of ovulation can alleviate negative premenstrual symptoms [12, 13, 14] and reduce anterior cruciate ligament (ACL) injury [15, 16], among others. For example, Hammarbäck et al. [13] showed that cyclical negative premenstrual symptoms such as irritability and breast tenderness disappear in anovulatory cycles. In addition, Yonkers et al. [14] found that administration of GnRH analogues, exogenous estrogen, and certain oral contraceptives at doses which inhibit ovulation is effective in reducing the symptoms. The paper [17] reported that ACL injuries in female athletes are significantly greater during the ovulatory phase and studies [18, 19, 15] suggested that oral contraceptive users have an almost 20% decreased risk for ACL injury.

- **Substantial evidence for its conclusions:** In its present form, the article describes an interesting mathematical exercise applying some results of control theory to a hyper-parameterized deterministic model. They deserve credit for this aspect. It is however based on both a series of strong assumptions more or less realistic and a very limited ideal "real" dataset which limits the possibility to translate these results to real contraceptive issues. Therefore, their conclusions in their formulation do not appear cautious enough to be shared to a wide audience who might be confused and impressed by the mathematical developments.

  **Response:** We agree with the reviewer that enough caution must be given in the interpretation of the results. Hence, as stated previously, we modified texts in the Discussion and Conclusion sections to emphasize the limitations of our study.

## References


[1] Harris L. Differential equation models for the hormonal regulation of the menstrual cycle [Ph.D. thesis]. North Carolina State University; 2002.

[2] Harris L, Selgrade J. Modeling endocrine regulation of the menstrual cycle using delay differential equations. Math Biosci. 2014;257:11–22.

[3] Wright A, FN G, Selgrade J, Olufsen M. Mechanistic model of hormonal contraception. PLOS Comp Biol. 2020 06;16(6):1–23.

[4] Margolskee A, Selgrade J. Dynamics and bifurcation of a model for hormonal control of the menstrual cycle with inhibin delay. Math Biosci. 2011;234(2):95–107.

[5] Welt CK, McNicholl D, Taylor A, Hall J. Female reproductive aging is marked by decreased secretion of dimeric inhibin. J Clin Endocrinol Metab. 1999;84(1):105–111.

[6] Obruca A, Korver T, Huber J, Killick S, Landgren BM, Struijs M. Ovarian function during and after treatment with the new progestagen Org 30659. Fertil steril. 2001;76(1):108–115.

[7] Deb S, Campbell B, Pincott-Allen C, Clewes J, Cumberpatch G, Raine-Fenning N. Quantifying effect of combined oral contraceptive pill on functional ovarian reserve as measured by serum anti-müllerian hormone and small antral follicle count using three-dimensional ultrasound. Ultrasound Obstetr Gynecol. 2012;39(5):574–580.

[8] Ordög T, Knobil E. Estradiol and the inhibition of hypothalamic gonadotropin-releasing hormone pulse generator activity in the rhesus monkey. Proceedings of the National Academy of Sciences. 1995;92(13):5813–5816.

[9] O'Byrne K, Knobil E. Electrophysiological approaches to gonadotrophin releasing hormone pulse generator activity in the rhesus monkey. Human Reproduction. 1993;8(suppl_2):37–40.

[10] Liu M, Liu S, Li L, Wang P, Li H, Li Y. LH levels may be used as an indicator for the time of antagonist administration in GnRH antagonist protocols—a proof-of-concept study. Frontiers in Endocrinology. 2019;10.

[11] Masello M, Ren Y, Erickson D, Giordano J. An automated controlled-release device for intravaginal hormone delivery. JDS communications. 2020;1(1):15–20.

[12] Guillebaud J. Contraception: your questions answered. 5th ed. Edinburgh, UK: Churchill Livingston; 2009.

[13] Hammarbäk S, Ekholm UB, Bäckström T. Spontaneous anovulation causing disappearance of cyclical symptoms in women with the premenstrual syndrome. Acta Endocrinol. 1991;125(2):132 – 137.

[14] Yonkers K, O'Brien P, Eriksson E. Premenstrual syndrome. The Lancet. 2008;371(9619):1200–1210.

[15] Herzberg SD, Motu'apuaka ML, Lambert W, Fu R, Brady J, Guise JM. The effect of menstrual cycle and contraceptives on ACL injuries and laxity: a systematic review and meta-analysis. Orthopaedic Journal of Sports Medicine. 2017;5(7):2325967117718781.

[16] Konopka JA, Hsue L, Chang W, Thio T, Dragoo JL. The effect of oral contraceptive hormones on anterior cruciate ligament strength. The American Journal of Sports Medicine. 2020;48(1):85–92.

[17] Wojtys EM, Huston LJ, Boynton MD, Spindler KP, Lindenfeld TN. The effect of the menstrual cycle on anterior cruciate ligament injuries in women as determined byhormone Levels. The American Journal of Sports Medicine. 2002;30(2):182–188.

[18] Gray AM, Gugala Z, Baillargeon JG. Effects of oral contraceptive use on anterior cruciate ligament injury epidemiology,. Medicine & Science in Sports & Exercise. 2016;48(4):648–654.

[19] Rahr-Wagner L, Thillemann TM, Mehnert F, Pedersen AB, Lind M. Is the use of oral contraceptives associated with operatively treated anterior cruciate ligament injury?: a case-control study from the Danish Knee Ligament Reconstruction Registry. The American Journal of Sports Medicine. 2014;42(12):2897–2905.
